# Associations of the HOMA2‐%B and HOMA2‐IR with progression to diabetes and glycaemic deterioration in young and middle‐aged Chinese

**DOI:** 10.1002/dmrr.3525

**Published:** 2022-03-08

**Authors:** Baoqi Fan, Hongjiang Wu, Mai Shi, Aimin Yang, Eric S. H. Lau, Claudia H. T. Tam, Dandan Mao, Cadmon K. P. Lim, Alice P. S. Kong, Ronald C. W. Ma, Elaine Chow, Andrea O. Y. Luk, Juliana C. N. Chan

**Affiliations:** ^1^ Department of Medicine and Therapeutics The Chinese University of Hong Kong Prince of Wales Hospital Hong Kong SAR China; ^2^ Hong Kong Institute of Diabetes and Obesity The Chinese University of Hong Kong Prince of Wales Hospital Hong Kong SAR China; ^3^ Li Ka Shing Institute of Health Sciences The Chinese University of Hong Kong Prince of Wales Hospital Hong Kong SAR China

**Keywords:** beta‐cell function, glycaemic deterioration, HOMA2, insulin resistance, T2D

## Abstract

**Aims:**

Insulin deficiency (ID) and resistance (IR) contribute to progression from normal glucose tolerance to diabetes to insulin requirement although their relative contributions in young‐onset diabetes is unknown.

**Methods:**

We examined the associations of HOMA2 using fasting plasma glucose and C‐peptide in Chinese aged 20–50 years with (1) progression to type 2 diabetes (T2D) in participants without diabetes in a community‐based cohort (1998–2013) and (2) glycaemic deterioration in patients with T2D in a clinic‐based cohort (1995–2014). We defined ID as HOMA2‐%B below median and insulin IR as HOMA2‐IR above median.

**Results:**

During 10‐year follow‐up, 62 (17.9%) of 347 community‐dwelling participants progressed to T2D. After 8.6 years, 291 (48.1%) of 609 patients with T2D had glycaemic deterioration. At baseline, progressors for T2D had higher HOMA2‐IR, while in patients with T2D, progressors for glycaemic deterioration had higher HOMA2‐IR and lower HOMA2‐%B than non‐progressors. The non‐ID/IR group and the ID/IR group had an adjusted odds ratios of 2.47 (95% CI: 1.28, 4.94) and 5.36 (2.26, 12.79), respectively, for incident T2D versus the ID/non‐IR group. In patients with T2D, 50% of the ID/IR group required insulin at 6.7 years versus around 11 years in the non‐ID/IR or ID/non‐IR, and more than 15 years in the non‐ID/non‐IR group. Compared with the latter group, the adjusted hazard ratios were 2.74 (1.80, 4.16) in the ID/non‐IR, 2.73 (1.78, 4.19) in the non‐ID/IR and 4.46 (2.87, 6.91) in the ID/IR group (*p*‐interaction = 0.049).

**Conclusions:**

In young Chinese adults, IR and ID contributed to progression to T2D and glycaemic deterioration.

## 1 INTRODUCTION

In 2019, 9.3% of adults aged 20–79 years were affected by diabetes with over 50% coming from Asia.^1^ Insulin resistance (IR), often due to obesity, dyslipidaemia and inflammation, and insulin deficiency (ID) both contribute to type 2 diabetes (T2D).^2^ However, the relative contributions of IR and ID to progression to T2D, especially amongst Asians who had both obese and non‐obese forms of diabetes, remained unclear.^3^ There are inter‐ethnic differences in phenotype between Asian and Caucasians with T2D.^3^, ^4^, ^5^ Compared with Caucasians, Asians had greater propensity for beta‐cell dysfunction.^6^ South Asians had more visceral adiposity and greater IR than East Asians who tend to have low body mass index (BMI) and low insulin secretion.^7^, ^8^ Within the same population (e.g. Koreans), phenotypic heterogeneity during different stages of disease progression might explain inconsistent reports with some researchers reporting the predominant role of decreased insulin sensitivity while others reporting the failure of insulin secretion to overcome IR during the development of diabetes.^9^, ^10^

Young age of diagnosis, body leanness and kidney disease are key features in Asian people with T2D. If not diagnosed and treated early, these young patients are exposed to decades of gluco‐lipotoxicity with high risk of premature disabilities and death.^11^ Despite the rising prevalence of young‐onset diabetes (YOD), the contributory roles of IR and ID had not been explored.^5^ In a population‐based survey of 94,952 Chinese aged 40 or above, the Homeostasis Model Assessment of insulin resistance had a larger effect size than the estimate of beta‐cell function in predicting progression to T2D.^12^ However, similar data in young Chinese adults are lacking. As different strategies (e.g. lifestyle modification, medications, surgery) may modulate ID and IR through different mechanisms of action,^5^, ^13^ elucidating the contributions of IR and ID in the progression to T2D in people without diabetes and progression to glycaemic deterioration in people with T2D might improve the precision of prognosis and intervention.

There are several methods for assessing beta‐cell function and insulin sensitivity. The original Homeostasis Model Assessment (HOMA) model proposed in 1985 was based on a mathematical feedback model first proposed in 1979.^14^ It is an estimate of beta‐cell function (%B) and insulin sensitivity (%S) during steady state calculated from fasting plasma glucose (FPG) and insulin levels. The original HOMA model correlates with estimations derived from clamp studies and can be used as surrogates in epidemiological studies and clinical practice.^15^, ^16^ In 1998, the updated HOMA model (HOMA2) was published to adjust for variations due to hepatic and peripheral glucose sensitivity, plasma glucose–insulin relationship for plasma glucose values above 180 mg/dl and plasma pro‐insulin levels. HOMA2 had been recalibrated to a normal population to set the reference value.^17^ They can be calculated using fasting C‐peptide (CP) levels instead of insulin, and had been shown to perform better than the original HOMA model in assessing IR or beta‐cell function as well as predicting progression to T2D.^18^, ^19^

In the late 1990s, we established multiple cohorts to ascertain the natural progression to T2D in Chinese in whom stored samples were available for the measurement of plasma CP levels. We selected subcohorts of young and middle‐aged Chinese to explore the associations of ID and IR estimated by HOMA2 with (1) progression to T2D in participants without T2D from a community‐based cohort; (2) glycaemic deterioration in patients with T2D from a clinic‐based cohort.

## 2 MATERIALS AND METHODS

### 2.1 Participants and study design

Hong Kong is a cosmopolitan city of 7.5 million people in southern China with rising incidence of YOD despite stabilizing and declining trends of diabetes in older age groups.^20^ This analysis used data from three prospective cohorts to ascertain the roles of IR and ID on progression to T2D and glycaemic deterioration in young adults. Cohorts are briefly described as below with details reported elsewhere.^21^, ^22^, ^23^

#### 2.1.1 BHBHK‐HKFDS cohorts: Progression to T2D

The “Better Health for Better Hong Kong” (BHBHK) is a community‐based cohort established during a territory‐wide health promotion and screening campaign targeting the workforce with a mean (SD) age of 42.4 (8.9) years and income close to or below the median salary in Hong Kong.^22^ Participants were randomly selected according to the distribution of occupational groups. A total of 11,965 invitations were sent and 4841 people (40.5%) responded and gave written consent. Of these, 561 participants were randomly selected to undergo detailed clinical and laboratory assessment at the Prince of Wales Hospital (PWH). The Hong Kong Family Diabetes Study (HKFDS) recruited first‐degree relatives (siblings and parents) of index patients with T2D, the majority of whom were diagnosed before 40 years old.^23^ Both cohorts were established in 1998–2003 to define the phenotypes for diabetes in young adults and were re‐evaluated in 2010–2014. All participants without known history of diabetes underwent 75 g oral glucose tolerance test (OGTT) to define the glycaemic status based on the American Diabetes Association (ADA) criteria.^24^ In this analysis, we included participants from BHBHK without diabetes at baseline who had follow‐up glycaemia status. We also randomly selected one participant without T2D at recruitment from each family in the Hong Kong Family Diabetes Study (HKFDS) to avoid confounding due to relatedness.

#### 2.1.2 HKDR: Glycaemic deterioration

The Hong Kong Diabetes Register (HKDR) is an ongoing quality improvement programme established in 1995 at the PWH, the CUHK‐affiliated teaching hospital.^21^ The HKDR consecutively enrolled 30–50 patients with physician‐diagnosed diabetes weekly referred to the PWH Diabetes Centre for comprehensive assessment of risk factors and complications. Referral sources included hospital‐based specialty clinics, community clinics, and private general practitioners. The HKDR was periodically linked to the territory‐wide electronic medical records with laboratory, prescription and hospital data. We selected enrollees between 1995 and 2014 with available data for analysis. We excluded patients with type 1 diabetes defined as positive glutamic acid decarboxylase antibody (>5 units/L) if data were available and patients with a history of diabetic ketoacidosis and/or continuous insulin treatment within 1 year of diagnosis. Patients treated with insulin at baseline were also excluded.

The selection criteria for BHBHK‐HKFDS and HKDR are shown in Figure S1. We limited our analysis to participants aged 20–50 years at recruitment with FPG of 3–25 mmol/L and fasting CP level of 0.2–3.5 nmol/L for using the HOMA2 calculator v2.2.3 (https://www.dtu.ox.ac.uk/homacalculator/). Participants with missing data for multivariable analysis were excluded. Only five participants from HKDR fulfilling the inclusion criteria were excluded due to missing data. In this analysis, we included 347 participants from BHBHK‐HKFDS and 609 patients from HKDR. Ethical approval was obtained from the Clinical Research Ethics Committees of the Chinese University of Hong Kong and all participants gave written informed consent.

### 2.2 Measurements

All participants underwent structured assessment at baseline including demographic status, medical history, clinical measurements and laboratory tests after an 8 h overnight fast. All participants eligible for analysis had measurements of FPG, HbA1c, CP, lipid profile (fasting total cholesterol [TC], triglycerides [TG], HDL‐C and calculated LDL‐C), estimated glomerular filtration rate (eGFR calculated by the Chronic Kidney Disease Epidemiology Collaboration equation)^25^ and random spot urinary albumin to creatinine ratio. All biochemical assays were performed by the Department of Chemical Pathology at the PWH with external accreditation. Plasma CP levels in the HKDR cohort were measured by Mercodia@ C‐peptide ELISA kit with lower detection limit of 25 pmol/L, within‐assay variation of <4.8% and total assay variation of <6.8%. Plasma CP levels in the BHBHK and HKFDS cohorts were measured by radioimmunoassay (Novo Nordisk, Copenhagen, Denmark) with lower detection limit of 0.1 nmol/L, an intra‐assay variation of 3.4% and an inter‐assay variation of 9.6%.^26^

### 2.3 Classification and definition of outcomes

#### 2.3.1 Progression to T2D in participants without diabetes

After excluding participants with diabetes at baseline, the glycaemic status of the BHBHK‐HKFDS cohort was ascertained from medical records, self‐report, or 75 g oral OGTT upon re‐evaluation in 2010–2014 using the ADA criteria (2009).^23^, ^24^ These criteria were used to define progressors and non‐progressors for T2D.

#### 2.3.2 Glycaemic deterioration in patients with T2D

Patients in the HKDR were censored on 30 June 2014. Glycaemic deterioration was defined by the composite outcome used in the IMI‐DIRECT study^27^: (1) continuous insulin treatment for ≥6 months or (2) failure of oral glucose lowering drugs (OGLDs) defined by two consecutive HbA1c ≥ 8.5% more than 3 months apart while on ≥2 OGLDs. Patients who reached this endpoint were defined as progressors for glycaemic deterioration. Follow‐up time was defined as the period between baseline visit and date of endpoint or censored date, whichever came first.

### 2.4 Statistical analyses

Data are presented as mean ± SD or median (interquartile range, IQR) as appropriate. For comparison, Student's *t*‐test, Mann–Whitney *U* test, Chi‐square (χ^2^), Fisher's exact test or Analysis of variance were used as appropriate. The *p* value for trend was calculated by linear regression of continuous variables on different groups of participants, or by Cochran–Armitage test for trend for categorical variables. We logarithmically transformed with base of two for HOMA2 analysed as continuous variables and stratified participants by median and tertile values of HOMA2 analysed as categorical variables. We defined ID as HOMA2‐%B below the median value and IR as HOMA2‐IR above the median value.

In the BHBHK‐HKFDS cohorts, we used logistic regression to assess associations of different combinations of IR and ID with the onset of T2D controlling for age, sex, BMI, TG/HDL‐C ratio, and family history of T2D which are considered as main confounders for progression to T2D. We further adjusted for glycaemic status (normal glucose tolerance [NGT] and prediabetes) and 1‐hour plasma glucose (PG) during 75 g OGTT, as an index of glucotoxicity to estimate the independent association of IR and ID with progression to T2D.^28^, ^29^

In the HKDR, we used restricted cubic spline regression with three knots (25th, 50th and 75th percentiles) to evaluate the shapes of relationships between HOMA2 and hazard ratios (HR) for glycaemic deterioration. We used Cox proportional hazard (PH) regression to estimate HR (95% CI) controlling for age, sex, diabetes duration, BMI, systolic blood pressure, HbA1c, TG/HDL‐C ratio and use of OGLDs. We used Schoenfeld residuals to assess the PH assumption. As the PH assumption was not met for diabetes duration and HbA1c, we stratified participants into the following categories: (1) duration: <1 year, 1–2 years, 3–5 years, >5 years; (2) glycaemic control: optimal (HbA1c < 7%), fair (HbA1c ≥ 7–9%) and poor (HbA1c ≥ 9%). Both disease duration and HbA1c categories were included as strata variables in the Cox models. No multicollinearity was detected by the variance inflation factor. We used the Kaplan–Meier estimator to estimate the median time to endpoint of glycaemic deterioration among different groups with log‐rank test for comparison.

Multiplicative interaction was detected by the likelihood ratio test. Additive interaction was evaluated by calculating the relative excess risk due to interaction, attributable proportion due to interaction (AP) and synergy index(S).^30^, ^31^ We used the Receiver Operating Characteristics (ROC) analysis to explore the utility of HOMA2 in predicting progression to T2D. The incremental improvement of prediction was detected by the Delong test.^32^ The discrimination of HOMA2 in survival analyses of glycaemic deterioration in T2D was assessed by the c‐index (concordance index) from the Cox model.^33^

All analyses were performed using the R software, version 3.6.3 (R Foundation for Statistical Computing). A two‐sided significance level of 0.05 was considered significant.

## 3 RESULTS

### 3.1 Characteristics of participants

In the BHBHK‐HKFDS cohort, 80% of 347 people without T2D were NGT at baseline (Table S1). After 10 years, 62 (17.9%) participants progressed to T2D. In the HKDR, 609 patients with T2D eligible for analysis were insulin‐naïve at baseline with a median (IQR) disease duration of two (1–4) years. After a median (IQR) follow‐up period of 8.6 (5.3–11.4) years, 291 (48.1%) patients progressed to glycaemic deterioration with an incidence rate of 57.26 (95% CI: 50.87, 64.23) per 1000 person‐years (Figure S1).

In this community‐ and clinic‐based cohort design including individuals in their 40s, we have identified four groups for analysis including progressors and non‐progressors for T2D in participants without diabetes, and progressors and non‐progressors for glycaemic deterioration in patients with T2D. The baseline data showed progressive decline in HOMA2‐%B and increase in HOMA2‐IR as the clinical status transited from NGT to incident diabetes and from diagnosed diabetes to glycaemic deterioration (*P*‐trend <0.001). Baseline beta‐cell function (HOMA2‐%B) and insulin sensitivity (HOMA2‐%S, the reciprocal of HOMA2‐IR) exhibited hyperbolic relationship in participants without diabetes but not in those with diabetes. Progressors for T2D and progressors for glycaemic deterioration had lower beta‐cell function for the same HOMA‐%S than their non‐progressor counterparts (Figure S2).

In the BHBHK‐HKFDS cohort, BMI, waist circumference (WC), TG and TG/HDL‐C ratio were positively associated while HDL‐C was inversely associated with HOMA2‐%B and HOMA2‐IR (Table S2). At baseline, progressors for T2D had higher CP, HOMA2‐IR, FPG, 2‐h PG, BMI, TC, LDL‐C, TG and lower HDL‐C than non‐progressors for T2D (Table 1).

**TABLE 1:** Baseline characteristics of Chinese young people classified as progressors and non‐progressors for T2D in the community‐based BHBHK‐HKFDS cohort and progressors and non‐progressors for glycaemic deterioration in patients with type 2 diabetes in the clinic‐based HKDR cohort

		Incident type 2 diabetes			Glycaemic deterioration			
		*Community‐based BHBHK‐HKFDS cohort in people without diabetes at baseline*			*Clinic‐based HKDR cohort in patients with type 2 diabetes*			*P* (trend) across 4 groups
		Non‐progressors	Progressors	*P* ^a^	Non‐progressors	Progressors	*P* ^b^	
Number		285	62		318	291		
Age (year)		39.97 (7.01)	40.94 (6.67)	0.321	42.45 (6.77)	41.68 (7.16)	0.175	<0.001
Men, n (%)		127 (44.6)	30 (48.4)	0.683	163 (51.3)	159 (54.6)	0.451	0.013
Duration of diabetes (year)		‐	‐	‐	1.00 [0.00, 3.00]	2.00 [1.00, 5.00]	<0.001	‐
Body mass index (kg/m^2^)		23.35 [21.24, 25.67]	25.39 [23.00, 28.13]	<0.001	25.39 [23.12, 28.32]	26.78 [23.97, 29.67]	0.001	<0.001
Waist circumference (cm)	Men	83.15 (7.59)	87.65 (8.04)	0.004	89.21 (9.49)	92.45 (10.41)	0.004	<0.001
	Women	74.57 (8.44)	80.33 (7.32)	<0.001	82.07 (9.60)	85.16 (11.20)	0.012	<0.001
Waist‐hip‐ratio		0.83 (0.07)	0.86 (0.06)	0.001	0.87 (0.06)	0.89 (0.06)	0.002	<0.001
Fasting plasma glucose (mmol/l)		4.85 (0.42)	5.34 (0.47)	<0.001	7.51 (2.37)	9.35 (3.28)	<0.001	<0.001
HbA1c (%)		‐	‐		6.8 (1.5)	7.9 (1.8)	<0.001	‐
HbA1c (mmol/mol)		‐	‐		51 (16)	63 (20)	<0.001	‐
Systolic blood pressure (mmHg)		114.71 (15.00)	125.04 (17.79)	<0.001	125.87 (16.88)	128.25 (14.88)	0.066	<0.001
Diastolic blood pressure (mmHg)		73.57 (10.16)	78.62 (12.28)	0.001	75.50 (10.46)	76.46 (9.76)	0.244	0.003
Total cholesterol (mmol/l)		5.05 (0.93)	5.26 (0.95)	0.113	4.97 (0.96)	5.18 (1.01)	0.009	0.382
Triglycerides (mmol/l)		0.96 [0.70, 1.43]	1.25 [0.84, 1.78]	0.001	1.40 [0.90, 1.96]	1.61 [1.15, 2.56]	<0.001	<0.001
HDL‐cholesterol (mmol/l)		1.53 (0.40)	1.40 (0.50)	0.027	1.33 (0.35)	1.27 (0.31)	0.012	<0.001
LDL‐cholesterol (mmol/l)		3.01 (0.86)	3.26 (0.83)	0.04	2.89 (0.87)	2.98 (0.84)	0.23	0.234
Ratio of TG to HDL‐C		0.65 [0.42, 1.10]	0.96 [0.57, 1.56]	0.001	1.13 [0.67, 1.64]	1.30 [0.83, 2.20]	<0.001	<0.001
Urinary ACR (mg/mmol/L)		0.64 [0.41, 1.24]	0.92 [0.55, 2.16]	0.004	0.82 [0.46, 2.56]	1.70 [0.70, 5.05]	<0.001	<0.001
eGFR (ml/min/1.73 m^2^)		‐	‐	‐	97.21 (17.44)	98.40 (17.67)	0.404	‐
Glycaemic status, n (%)	NGT	249 (87.4)	27 (43.5)	<0.001	‐	‐	‐	‐
	Isolated IFG	14 (4.9)	10 (16.1)		‐	‐		
	Isolated IGT	20 (7.0)	12 (19.4)		‐	‐		
	IFG and IGT	2 (0.7)	13 (21.0)		‐	‐		
Use of glucose lowering oral drugs, *n* (%)		‐	‐	‐	209 (65.7)	239 (82.1)	<0.001	‐
Use of lipid lowering drugs, *n* (%)		‐	‐	‐	47 (14.8)	45 (15.5)	0.903	‐
Use of BP lowering drugs, *n* (%)		‐	‐	‐	100 (31.4)	98 (33.7)	0.617	‐
Use of RAS inhibitors, *n* (%)		‐	‐	‐	52 (16.6)	63 (22.1)	0.106	‐
C‐peptide (pmol/L)		353.20 [273.45, 491.71]	480.99 [364.41, 619.13]	<0.001	590.87 [419.52, 837.93]	647.77 [437.06, 873.81]	0.118	<0.001
HOMA2‐%B		84.30 [72.00, 111.50]	85.10 [75.62, 100.75]	0.593	62.15 [39.50, 87.33]	45.10 [25.55, 71.55]	<0.001	<0.001
HOMA2‐IR		0.77 [0.60, 1.06]	1.06 [0.81, 1.38]	<0.001	1.47 [1.02, 2.12]	1.75 [1.19, 2.42]	<0.001	<0.001
1‐h plasma glucose (mmol/L)		7.93 (2.21)	10.46 (2.17)	<0.001	**‐**	**‐**	**‐**	**‐**
2‐h plasma glucose (mmol/L)		5.81 (1.53)	7.17 (1.87)	<0.001	**‐**	**‐**	**‐**	**‐**

*Note*: Data are expressed as mean (SD) or number (%) median [IQR].

Abbreviations: ACR, albumin creatinine ratio; BHBHK‐HKFDS, Better Health for Better Hong Kong – Hong Kong Family Diabetes Study; BP, blood pressure; eGFR, estimated glomerular filtration rate; HKDR, Hong Kong Diabetes Register; IFG, impaired fasting glucose; IGT, impaired glucose tolerance; NGT, normal glucose tolerance; RAS, renin angiotensin system; TG, triglycerides.

^a^ *P* indicates significance for comparison between progressors and non‐progressors for T2D.

^b^ *P* indicates significance for comparison between progressors and non‐progressors for glycaemic deterioration; *P* (trend) indicates significance for a linear trend across four groups of participants from non‐progression to T2D to glycaemic deterioration in T2D.

In the HKDR cohort, BMI and WC were positively associated while HbA1c and TC were inversely associated with HOMA2‐%B (Table S2). There were positive associations of most cardio‐metabolic risk factors (except HDL‐C) with HOMA2‐IR (Table S2). At baseline, progressors for glycaemic deterioration had lower HOMA2‐%B and higher HOMA2‐IR but similar CP levels as the non‐progressors (Table 1). They also had longer disease duration, higher HbA1c, BMI, and worse lipids (Table 1).

There were gender differences in several metabolic factors at baseline in both cohorts (Table S3) but there was no interaction between HOMA2 and gender for the associations with T2D and glycaemic deterioration.

### 3.2 Association of HOMA2 with progression to T2D

**FIGURE 1:**
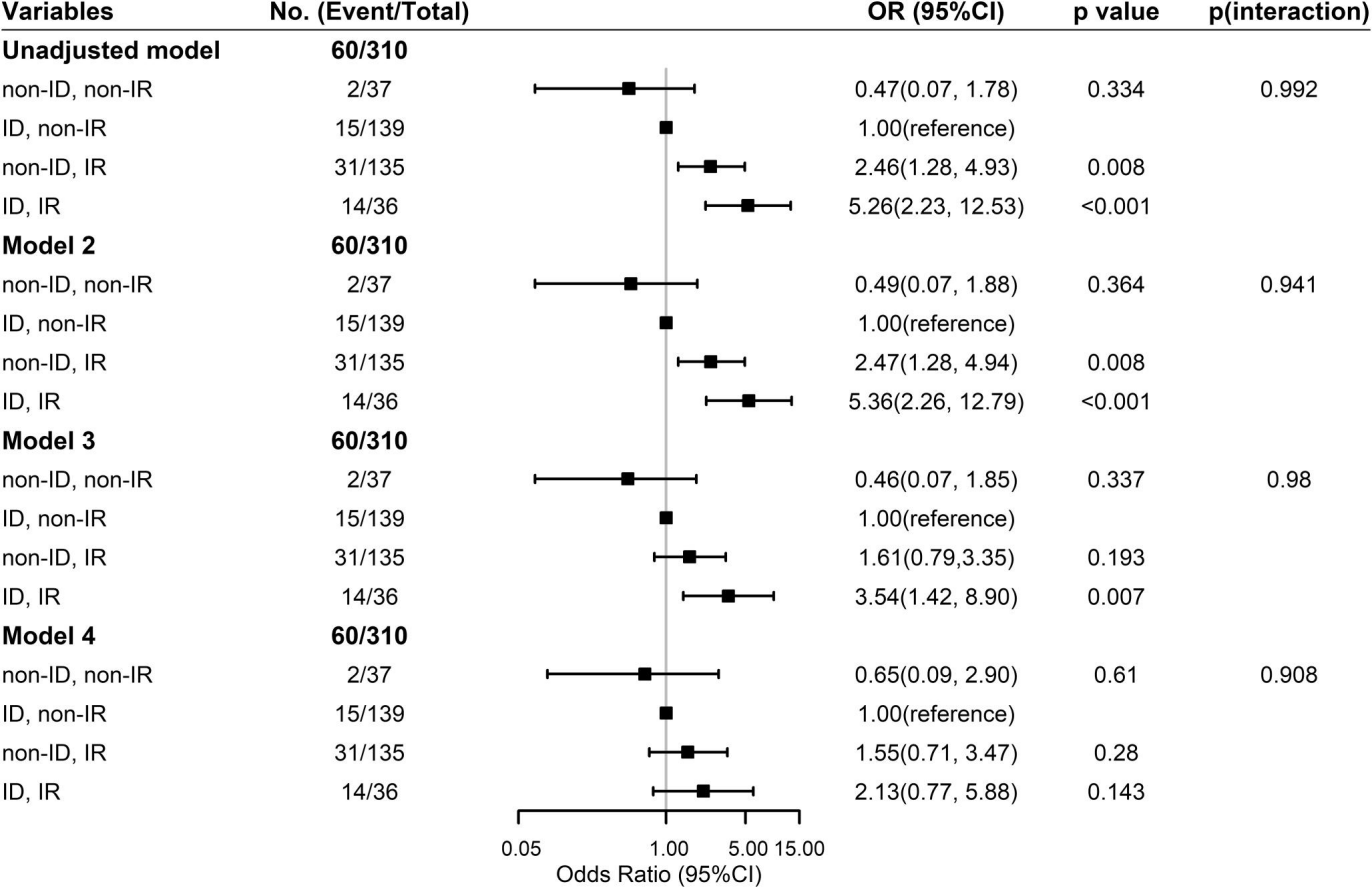
**Associations of HOMA2 indexes with progression to type 2 diabetes (T2D) in people without diabetes.** ^a^Insulin deficiency (ID) was defined as HOMA2‐%B below median value and insulin resistance (IR) as HOMA2‐IR above median value. ^b^Due to the small number of participants in the non‐ID/non‐IR group with incident T2D (*n* = 2), we used the ID/non‐IR group as reference group. Model 2 was adjusted for age, sex; model 3 was adjusted for variables in model 2 plus BMI, ln (triglycerides to HDL‐C ratio) and family history of T2D; model 4 was adjusted for variables in model 3 plus glycaemic status at baseline and plasma glucose at 60 min during 75 g oral glucose tolerance. The horizontal axis for odds ratios is expressed on a natural logarithmic scale. ^c^ *P*(interaction) was calculated for multiplicative interaction by likelihood ratio test

High HOMA2‐IR and low HOMA2‐%B were individually associated with increased odds of progression to T2D with HOMA2‐IR showing greater effect size after adjusting for covariates including glycaemic status (NGT, prediabetes) (Table S4). Due to the small number of participants in the non‐ID/non‐IR group with incident T2D (*n* = 2), we used the ID/non‐IR group as the reference group. Compared with the ID/non‐IR group, the non‐ID/IR had an OR of 2.46 (95% CI: 1.28, 4.93) and the ID/IR group had an OR of 5.26 (95% CI: 2.23, 12.53) for progression to T2D (Figure 1). The associations remained significant after adjusting for age and sex, but progressively attenuated after adjusting for BMI, TG/HDL‐C ratio, and family history of T2D. This was rendered non‐significant after further adjusting for FPG and 1 h PG during 75 g OGTT. There were no significant multiplicative or additive interactions between IR and ID on progression to T2D (Figure 1, Figure S3 and Table S5).

### 3.3 Association of high HOMA2‐IR and low HOMA2‐%B with glycaemic deterioration in T2D

**FIGURE 2:**
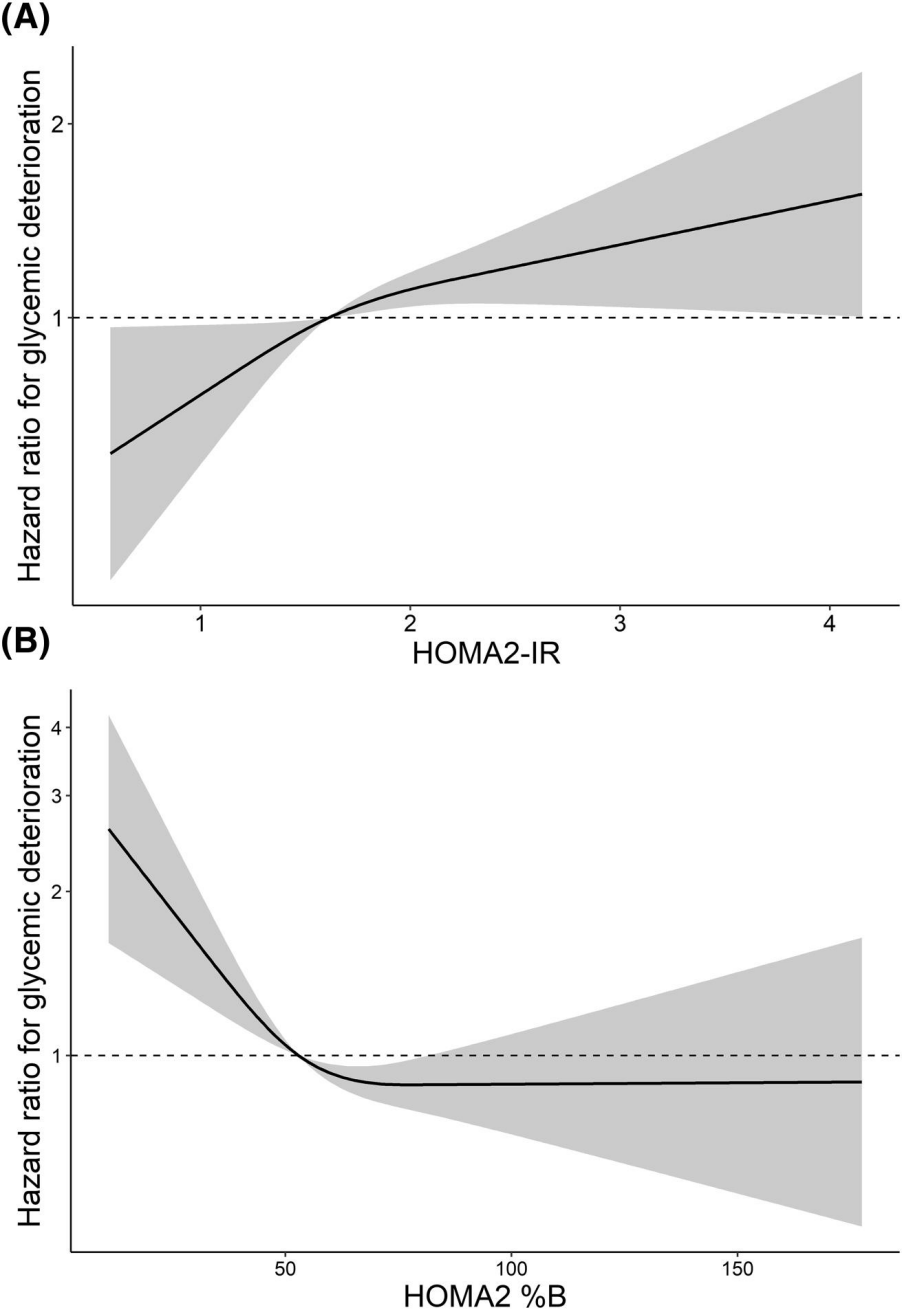
**Restricted cubic spline analysis of HOMA2‐IR** (A) **and HOMA2‐%B** (B) **on hazard ratios (HR) of progression to glycaemic deterioration.** ^a^Spline analyses were adjusted for age, sex, ln (TG/HDL‐C), body mass index, systolic blood pressure, baseline treatment of oral glucose lowering drugs, and strata by diabetes duration and HbA1c. ^b^For Figure 3A, *P* (overall) = 0.240, *P* (non‐linearity) = 0.998; for Figure 3B, *P* (non‐linearity) = 0.009

In the HKDR, there was a linear and a reverse J‐shaped association between HOMA2‐IR (*P*‐overall = 0.240, *P*‐non‐linearity = 0.998, Figure 2A) and HOMA2‐%B (*P*‐non‐linearity = 0.009, Figure 2B) with the risk of glycaemic deterioration, respectively. The HR declined linearly until the 50^th^ percentile of HOMA2‐%B and remained flat thereafter (Figure 2B). Below the median (52.90), every one unit decline in HOMA2‐%B was associated with an increase of HR by 1.02 (95% CI: 1.00–1.03) with full adjustment (Data not shown).

**FIGURE 3:**
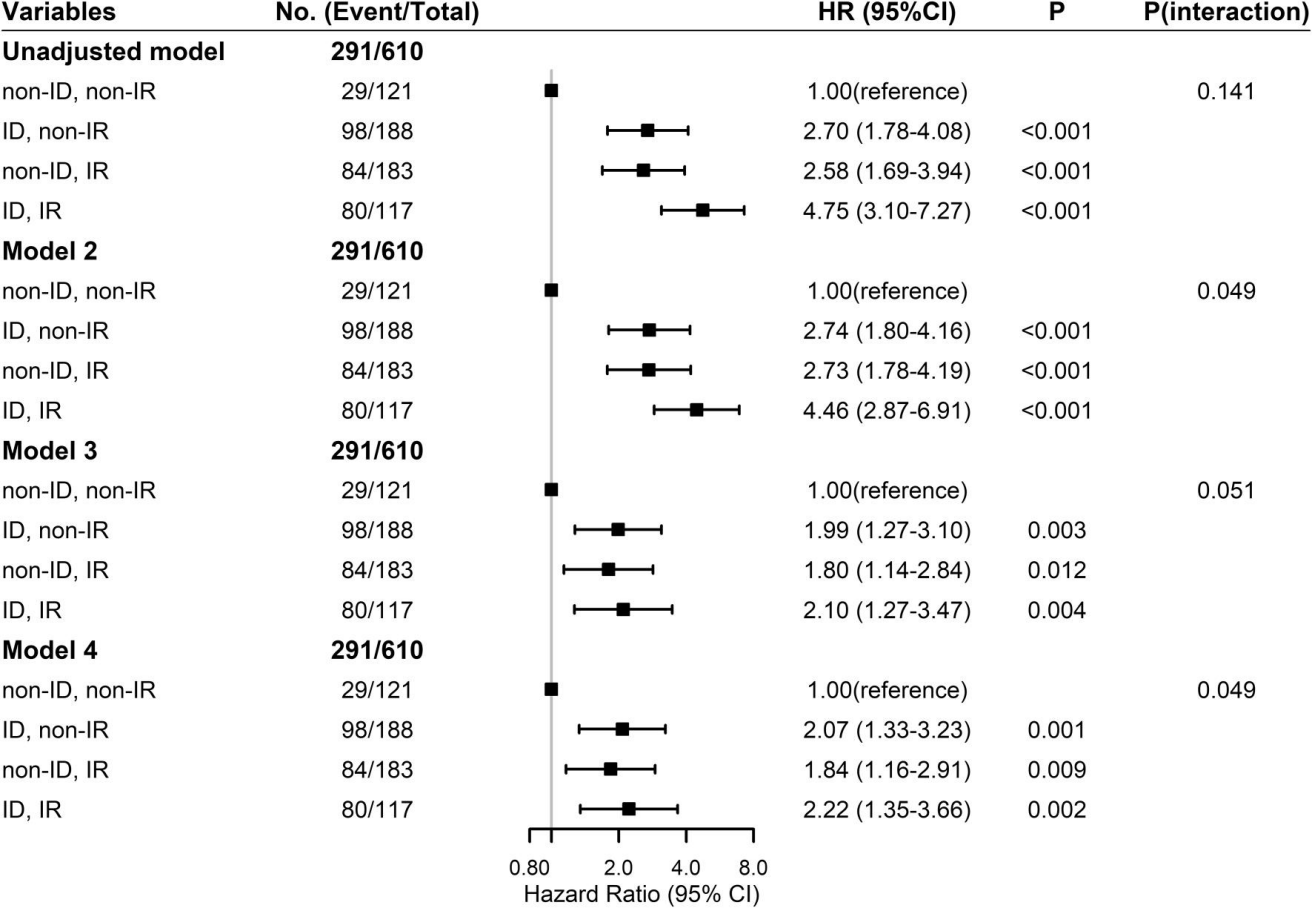
**Associations of HOMA2 indexes with progression to glycaemic deterioration in patients with T2D.** ^a^Insulin deficiency (ID) was defined as HOMA2‐%B below median value and insulin resistance (IR) as HOMA2‐IR above median value. ^b^We used the non‐ID/non‐IR group as reference, model 2 was adjusted for age, sex, and strata by duration of diabetes; model 3 was adjusted for variables in model 2 plus ln (triglycerides to HDL‐C ratio), BMI, systolic blood pressure, and strata by HbA1c; model 4 was adjusted for variables in model 3 and baseline use of oral glucose lowering drugs. The horizontal axis for hazard ratios is expressed on a natural logarithmic scale. ^c^ *P*(interaction) was calculated for multiplicative interaction by likelihood ratio test

**FIGURE 4:**
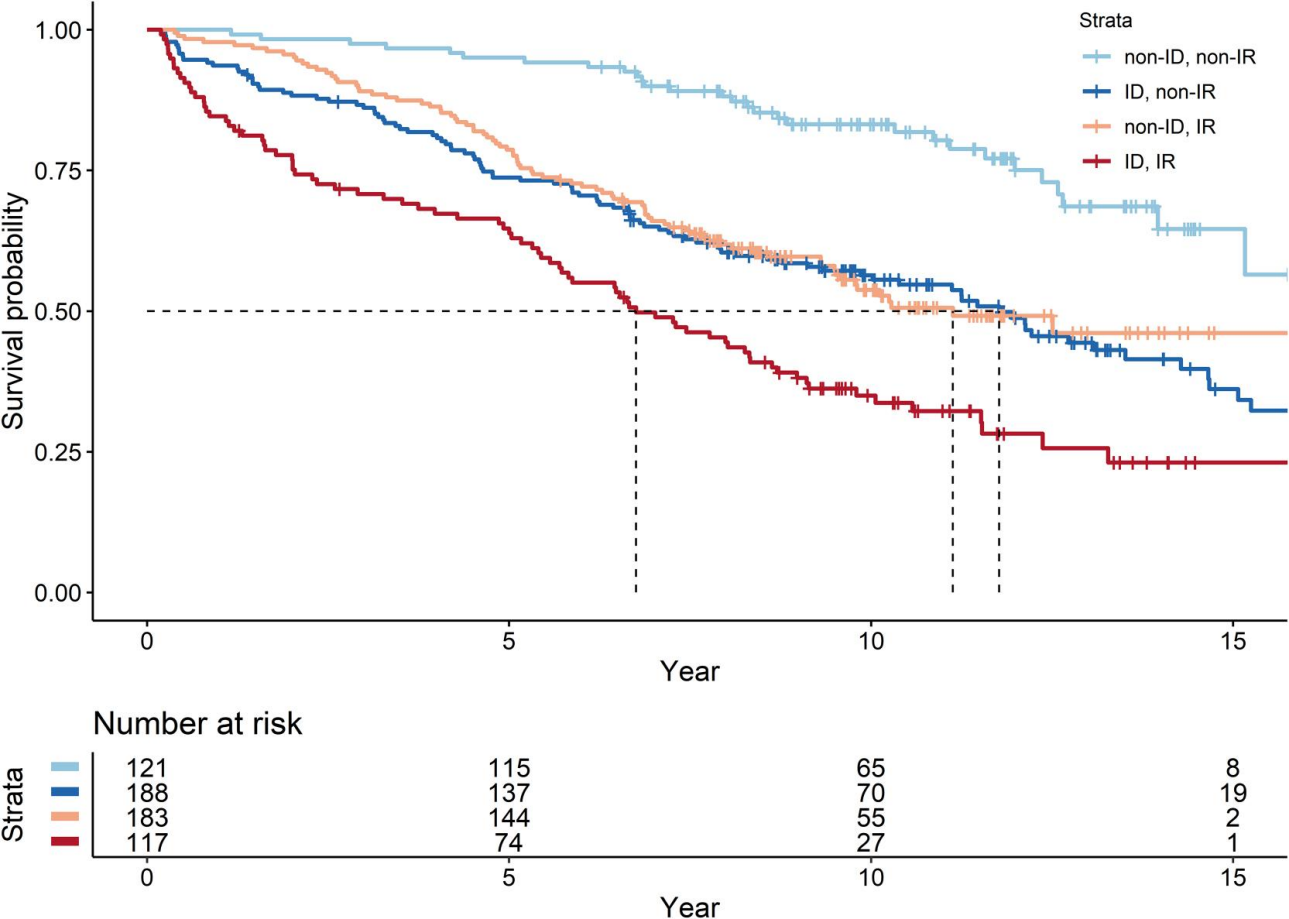
**Kaplan–Meier curves of glycaemic deterioration stratified by the median of HOMA2‐%B and HOMA2‐IR in young Chinese patients with type 2 diabetes.** ^a^Strata: non‐ID, non‐IR: HOMA2‐%B above the median and HOMA2‐IR below the median; Insulin deficiency (ID), non‐IR: HOMA2‐%B below the median and HOMA2‐IR below the median; non‐ID, IR: HOMA2‐%B above the median and HOMA2‐IR above the median; ID, IR: HOMA2‐%B below the median and HOMA2‐IR above the median. *P (log rank) < 0.0001

After full adjustment, the ID group had a HR of 1.64 (95% CI: 1.21–2.22) for glycaemic deterioration compared with the non‐ID and the IR group had a HR of 1.40 (95% CI: 1.05–1.88) compared with the non‐IR (Table S6). Compared with the non‐ID/non‐IR group, the ID/non‐IR [HR: 2.74 (1.80, 4.16)] and non‐ID/IR [HR: 2.73 (1.78, 4.19)] groups had more than 2.5‐fold higher risk of glycaemic deterioration whereas the ID/IR group had a HR of 4.46 (95% CI: 2.87, 6.91) (Figure 3). These associations were attenuated but remained significant after adjusting for glycaemic and lipid profiles, baseline treatment of OGLDs (Figure 3) and renal function (data not shown). There was multiplicative interaction between HOMA2‐%B and HOMA2‐IR on progression to glycaemic deterioration (*p* = 0.049 in the fully adjusted model, Figure 3). In the Kaplan–Meier analysis, 50% of patients progressed to glycaemic deterioration within 7 years in the ID/IR group compared with around 11 years in those with either ID or IR only and more than 15 years in the non‐ID/non‐IR group (Figure 4).

### 3.4 Discrimination of HOMA2 for progression to T2D and glycaemic deterioration

We explored the power of using HOMA2‐IR and HOMA2‐%B to discriminate between progressors and non‐progressors for T2D as well as progressors and non‐progressors for glycaemic deterioration in T2D. Analysed as a continuous variable, log2(HOMA2‐%B) (incremental change (Δ) of area under the curve (AUC): 0.074, *p* < 0.001, Figure S6A) and log2(HOMA2‐IR) (incremental change (Δ) AUC: 0.081, *p* = 0.002, Figure S6B) both showed incremental discriminative effect in predicting progression to T2D on top of age, sex, cardio‐metabolic risk factors, and family history of diabetes. In the HKDR, HOMA2‐%B improved the discrimination for glycaemic deterioration (*c*‐index (95% CI): 0.628 (0.589, 0.667) without log2(HOMA2‐%B) versus 0.655 (0.616, 0.694) with log2(HOMA2‐%B)). For HOMA2‐IR, the respective *c*‐index values were 0.642 (0.603, 0.681) versus 0.655 (0.615, 0.694).

## 4 DISCUSSION

Young‐onset diabetes is a heterogenous condition with poor outcomes.^11^ Defining the relative contributions of IR and ID on disease trajectory may inform practice and policies. We used three prospective cohorts of adults aged 18–50 years to explore the contributions of ID and IR on risks of incident T2D and glycaemic deterioration. Using median values of HOMA2 to codify IR and ID status, in participants without diabetes, the ID/IR group had 5.4‐fold increased risk of incident T2D versus the ID/non‐IR group. In patients with T2D, the ID/IR group had 4.5‐fold increased risk of glycaemic deterioration versus the non‐ID/non‐IR group. They were attenuated but remained significant after controlling for obesity, lipid and glucose indexes. While there was a linear relationship between HOMA2‐IR and glycaemic deterioration, the relationship of HOMA2‐%B was J‐shaped with values below the median being linearly associated with increased risk of glycaemic deterioration.

Homeostasis model assessment had been widely used to predict risks of T2D and metabolic syndrome, albeit similar data are sparse for the updated HOMA2 and in Chinese young adults. Compared with the original HOMA model, HOMA2 had better performance in predicting incident T2D.^18^, ^19^ C peptide is produced in the same amount as insulin and considered a good measure of endogenous insulin secretion even in insulin‐treated patients. In a prospective population‐based cohort in Korea including 104,694 individuals with age distribution (38.9 ± 7.4 years in NGT and prediabetes) similar to our cohorts, researchers used the same criteria to define diabetes and reported that both HOMA2‐IR and HOMA2‐%B predicted incident T2D.^18^ While different study design and assays do not allow direct comparisons, Korean participants^18^ appeared to have higher HOMA2‐IR than our Chinese participants (1.27 ± 0.45 vs. 0.87 ± 0.35 in NGT, 1.46 ± 0.55 vs. 1.08 ± 0.43 in prediabetes) with similar trends for HOMA2‐%B (100.09 ± 35.51 vs. 94.11 ± 28.4 in NGT, 93.14 ± 31.67 vs. 85.14 ± 28.67 in prediabetes) and TG (1.30 ± 0.83 vs. 1.23 ± 0.85 in NGT, 1.59 ± 1.04 vs. 1.39 ± 0.98 in prediabetes) (Table S1). These differences might be attributable to ecological, environmental, lifestyle and other factors which can modulate phenotypes and disease trajectories.

In our study, HOMA2‐IR predicted incident T2D independent of baseline glycaemic status (NGT, prediabetes) and 1 h PG. This finding is similar to other reports from Iranian (aged 20–86 years, using HOMA2) and Chinese (aged above 40, using HOMA1) cohorts, supporting the important role of IR mediated by obesity and glucolipotoxicity in progression to T2D in both young and older Chinese people.^12^, ^34^ Given the lower beta‐cell capacity in Asians compared with Caucasians, the coexistence of IR might accelerate the loss of beta‐cell dysfunction leading to T2D.^4^ Besides, dyslipidaemia characterised by low HDL‐C was reported to increase T2D risk through promoting beta‐cell dysfunction.^35^ Identifying individuals with beta‐cell dysfunction for optimising control of glucose, lipid and body weight may retard the progression to T2D.^5^

To our knowledge, there was no published data using HOMA2 to predict glycaemic deterioration in T2D. Such information is particularly relevant to young patients, given their long disease duration, and the identification of any modifiers that delay insulin requirement will inform clinical management. The reverse J‐shaped relationship between HOMA2‐%B and HRs of glycaemic deterioration suggested a threshold value where glycaemic deterioration accelerated. The HR decreased with increasing HOMA2‐%B values up to the median and remained flat thereafter. In contrast to progression to T2D where IR plays a more important role, in patients with T2D, ID showed greater effect size than IR on glycaemic progression. These findings accorded with that from the United Kingdom Prospective Diabetes Study and the Belfast Diet Study.^36^, ^37^ In White people with T2D, baseline HbA1c, young age, and weight gain independently predicted glycaemic deterioration (HbA1c ≥ 7% or initiation of GLDs).^38^ Apart from these aforementioned factors, we previously reported in the expanded HKDR cohort including Chinese patients with T2D and across all ages that triglycerides, smoking and microvascular complications were also associated with glycaemic deterioration. We further found an independent association between a polygenic risk score consisting of variants associated with beta‐cell function but not obesity with glycaemic deterioration.^39^ In this study of young and middle‐aged Chinese with T2D, HOMA2‐%B predicted glycaemic deterioration with nominally incremental values after adjusting for other risk factors. Taken together, while IR, mediated mainly by obesity and dyslipidaemia may hasten glycaemic deterioration, beta‐cell dysfunctions due to genetic factors are an important consideration. To this end, at baseline, the HOMA2‐%B of patients with diagnosed T2D was only 50% that of similar‐aged individuals without diabetes in the community‐based cohort, in support of the importance of beta cell dysfunction at the onset of diabetes.

Importantly, we identified multiplicative interactions where IR conferred higher risk of progression to glycaemic deterioration in patients without ID than those with ID (Figure S5). Similarly, non‐ID patients without IR had more durable glycaemic control than non‐ID patients with IR. These interactive effects between IR and ID have therapeutic implications. Thus, in patients with T2D, correcting ID using insulin‐secretagogues may not delay glycaemic durability if IR coexists. Similarly, correcting IR by weight reduction or using insulin sensitizers may not delay progression if ID coexists. In other words, if ID and IR coexist, these metabolic defects need to be addressed simultaneously in order to maximise the effects of intervention calling for more precise definition of metabolic status. In the VERIFY Study, newly diagnosed patients with T2D initiated with metformin and vildagliptin (a dipeptidyl‐peptidase‐4 inhibitor) combination therapy which corrected both ID and IR had better glycaemic durability than patients treated with metformin monotherapy followed by addition of vildagliptin only when glycaemic control worsened.^40^

In the HKDR, the median disease duration at enrolment was 1 year in non‐progressors for glycaemic deterioration and 2 years among the progressors. Despite this short disease duration, HOMA2‐%B of progressors for glycaemic deterioration was 50%–70% while that of HOMA2‐IR was 2‐fold compared with non‐progressors. Some experts proposed the use of C‐peptide <200 pmol/L to identify patients with ID such as those with latent autoimmune diabetes in adults or monogenic diabetes.^41^ However, these CP values can be confounded by hyperglycaemia as evident by the high (>200 pmol/L) and/or similar CP levels between progressors and non‐progressors for glycaemic deterioration in our cohort. At baseline, despite having similar fasting CP levels, progressors for glycaemic deterioration had 30% lower HOMA2‐%B than non‐progressors. On ROC analysis, we also demonstrated the incremental value of HOMA2 in predicting progression to T2D and glycaemic deterioration although external validation in similar cohorts is required.

To our knowledge, this is the first report on the contributory roles of ID and IR on the development and progression of T2D in young people which is a rising healthcare burden.^5^ Ideally, the analysis should be conducted in a prospective cohort spanning from NGT to diabetes to insulin requirement although this would require a large sample size with long follow‐up period and enough events for both outcomes. In this study, we have curated cohorts observed in similar settings with detailed and comprehensive documentation of characteristics at baseline with complementary outcomes of interest. Although our results have potential utilities for prognostication and intervention, our study also had limitations. Firstly, the sample size was relatively small due to selection criteria, causing the wide confidence interval for the estimates of associations between HOMA2 and outcomes. Secondly, the HKFDS cohort included family members of patients with YOD although only one individual was selected from each family with adjustment for family history in the multivariable models. Thirdly, HOMA index are indirect measures of ID and IR in steady state after overnight fast, although their validity had been confirmed during dynamic tests.^15^, ^16^, ^42^ The use of GLDs might influence the accuracy of HOMA. However, none of our patients were treated with insulin and we have adjusted for baseline use of OGLDs in the multivariable models. Our results might apply only to Chinese young and middle‐aged adults although similar findings had been reported in other Asian populations.

In conclusion, in Chinese young and middle‐aged adults, obesity and dyslipidaemia were closely associated with HOMA2‐IR which predicted progression to T2D. Beta‐cell dysfunction estimated by HOMA2‐%B was a stronger predictor than HOMA2‐IR for glycaemic deterioration in patients with T2D. People with both IR and ID had the highest risk for progression to T2D and glycaemic deterioration, with ID and IR exhibiting multiplicative interactions in glycaemic deterioration. Pending validation in larger sample size, our results supported the use of HOMA2 to define the differential roles of ID and IR to inform prevention strategies and clinical practice.

## CONFLICT OF INTEREST

Juliana C. N. Chan has received research grants and/or honoraria for consultancy and/or giving lectures from AstraZeneca, Bayer, Boehringer Ingelheim, Celltrion, Eli‐Lilly, Hua Medicine, Lee Powder, Merck Serono, Merck Sharp & Dohme, Pfizer, Servier, Sanofi and Viatris. Andrea O. Y. Luk has served as an advisory committee member for AstraZeneca, Boehringer Ingelheim, Sanofi and Amgen and has received research grants and travel grants from AstraZeneca, Boehringer Ingelheim, MSD, Novartis, Novo Nordisk, Sanofi and Amgen. Ronald C. W.Ma has received research grants for clinical trials from AstraZeneca, Bayer, MSD, Novo Nordisk, Sanofi, Tricida and honoraria for consultancy or lectures from AstraZeneca, and Boehringer Ingelheim. Alice P. S. Kong has received research grants and/or speaker honoraria from Abbott, Astra Zeneca, Bayer, Boehringer Ingelheim, Eli‐Lilly, Merck Serono, Nestle, Novo Nordisk and Sanofi. All other authors declare that they have no competing interests.

## ETHICS STATEMENT

The study was approved by the Chinese University of Hong Kong New Territories East Cluster Clinical Research Ethics Committee.

## AUTHOR CONTRIBUTIONS

Baoqi Fan contributed to the statistical analysis, results interpretation, drafting and revising the manuscript critically, and approved the final version. Hongjiang Wu, Mai Shi, Aimin Yang, Eric S.H. Lau, Claudia H.T. Tam, Dandan Mao and Cadmon K.P. Lim contributed to results interpretation, revised the manuscript critically and approved the final version. Alice P.S. Kong and Ronald C.W. Ma revised the manuscript critically and approved the final version. Elaine Chow and Andrea O.Y. Luk contributed to the results interpretation, revised the manuscript critically and approved the final version. Juliana C.N. Chan contributed to the conception of the article, statistical analysis and results interpretation, drafting of the manuscript and revising the manuscript critically, and approved the final version. All authors believe that the manuscript represented honest work, contributed meaningfully to this manuscript and approved the final version. Juliana C.N. Chan is the guarantor of this work, has full access to all the data in the study and takes responsibility for the integrity of the data and the accuracy of the data analysis.

### PEER REVIEW

The peer review history for this article is available at https://publons.com/publon/10.1002/dmrr.3525.

## Supporting information

Supporting Information 1

## Data Availability

Consent had not been obtained from study participants for data sharing in public domain but are available from the corresponding author upon reasonable request. Supplementary tables and figures are available online.
