# Supplementary material for: Associations of the HOMA2‐%B and HOMA2‐IR with progression to diabetes and glycaemic deterioration in young and middle‐aged Chinese
Source: Diabetes Metab Res Rev. 2022 Mar 8;38(5):e3525. doi: 10.1002/dmrr.3525 (PMC9542522; doi:10.1002/dmrr.3525)
Supplement: Supplementary file 1 — Supporting Information 1 [file DMRR-38-e3525-s001.docx]

**Supplementary Appendix**

Associations of the HOMA2-%B and HOMA2-IR with Progression to Diabetes and Glycaemic Deterioration in Young and Middle-aged Chinese

Manuscript number: DMRR-21-RA-527

| **Supplement Tables** | |  |
| --- | --- | --- |
| Table S1 | Baseline characteristics in normal subjects from the BHBHK-HKFDS and patients with T2D from HKDR stratified by their glycaemic status at baseline .................................................................................................................... | 2 |
| Table S2 | Associations between cardio-metabolic factors and HOMA2 indices in subjects with or without diabetes at baseline ........................................................................ | 4 |
| Table S3 | Baseline characteristics of participants stratified by gender in community-based BHBHK-HKFDS and clinic-based HKDR cohorts ................................................ | 5 |
| Table S4 | Logistic regression of HOMA2-%B and HOMA2-IR for progression to type 2 diabetes .................................................................................................................... | 7 |
| Table S5 | Statistics of detection of additive interaction between HOMA2 %B and HOMA2 IR in the association with progression to type 2 diabetes and glycemic deterioration............................................................................................................. | 8 |
| Table S6 | Cox regression of HOMA2-%B and HOMA2-IR for glycemic deterioration ............................................................................................................ | 9 |
| **Supplement Figures** | |  |
| Figure S1 | Flowchart of cohorts selection …………………………………………………… | 10 |
| Figure S2 | Relationship between baseline HOMA2-%B and HOMA2-%S in progressors versus non-progressors for type 2 diabetes (T2D) or glycaemic deterioration........ | 11 |
| Figure S3 | Logistic regression for progression to type 2 diabetes stratified by the median values of HOMA2 %B and HOMA2 IR respectively ............................................. | 12 |
| Figure S4 | Survival curve of glycemic deterioration stratified by median or tertile values of HOMA2 %B and HOMA2-IR ................................................................................ | 13 |
| Figure S5 | Cox regression for glycemic deterioration stratified by the median value of HOMA2 %B and HOMA2 IR respectively ............................................................ | 14 |
| Figure S6 | Receiver Operating Characteristic Curve of HOMA2 indexes on onset of type 2 diabetes and progression to insulin requirement in patients with type 2 diabetes .. | 15 |

**Supplement Tables**

Table S1. Baseline characteristics in normal subjects from the BHBHK-HKFDS and patients with T2D from HKDR stratified by their glycaemic status at baseline

|  | | **BHBHK-HKFDS** | | **HKDR** |  |  |
| --- | --- | --- | --- | --- | --- | --- |
|  | | **NGT** | **prediabetes** | **Type 2 diabetes** | ***P*^*^** | ***P*^†^** |
| Number | | 276 | 71 | 609 |  |  |
| Age (years) | | 39.66 (7.19) | 42.00 (5.57) | 42.08 (6.97) | 0.011 | <0.001 |
| Sex (%) | Men | 126 (45.7) | 31 (43.7) | 322 (52.9) | 0.868 | 0.073 |
|  | Women | 150 (54.3) | 40 (56.3) | 287 (47.1) |  |  |
| Duration of diabetes (year) | | - | - | 2.00 [1.00, 4.00] | - | - |
| Body mass index (kg/m2) | | 23.44 [21.39, 25.56] | 25.24 [22.38, 28.08] | 25.93 [23.53, 29.02] | 0.001 | <0.001 |
| Waist circumference (cm) | Men | 83.37 (7.72) | 86.61 (8.00) | 90.81 (10.07) | 0.039 | <0.001 |
|  | Women | 74.77 (8.15) | 78.44 (9.31) | 83.49 (10.46) | 0.015 | <0.001 |
| Waist-hip-ratio | | 0.83 (0.07) | 0.85 (0.07) | 0.88 (0.06) | 0.03 | <0.001 |
| Fasting plasma glucose (mmol/l) | | 4.82 (0.36) | 5.42 (0.54) | 8.39 (2.99) | <0.001 | <0.001 |
| HbA1c (%) | | - | - | 7.32 (1.75) | - | - |
| HbA1c (mmol/mol) | | - | - | 57 (19) | - | - |
| Systolic blood pressure (mmHg) | | 114.55 (15.43) | 124.38 (15.91) | 127.01 (15.99) | <0.001 | <0.001 |
| Diastolic blood pressure (mmHg) | | 73.24 (10.36) | 79.28 (10.85) | 75.96 (10.13) | <0.001 | <0.001 |
| Total cholesterol (mmol/l) | | 5.05 (0.91) | 5.24 (1.01) | 5.07 (0.99) | 0.136 | 0.336 |
| Triglycerides (mmol/l) | | 0.96 [0.72, 1.52] | 1.16 [0.80, 1.64] | 1.50 [1.03, 2.26] | 0.077 | <0.001 |
| HDL-cholesterol (mmol/l) | | 1.52 (0.44) | 1.42 (0.34) | 1.30 (0.33) | 0.054 | <0.001 |
| LDL-cholesterol (mmol/l) | | 3.01 (0.85) | 3.24 (0.90) | 2.93 (0.85) | 0.044 | 0.013 |
| Ratio of TG to HDLC | | 0.64 [0.42, 1.16] | 0.78 [0.55, 1.24] | 1.20 [0.74, 2.00] | 0.053 | <0.001 |
| Urinary ACR (mg/mmol) | | 0.64 [0.42, 1.24] | 0.85 [0.52, 1.80] | 1.19 [0.54, 3.60] | 0.007 | <0.001 |
| eGFR (ml/min/1.73 m^2^) | | - | - | 97.78 (17.55) | - | - |
| Glycaemic status (%) | NGT | 276 (100.0) | 0 (0.0) | - | <0.001 | - |
|  | Isolated IFG | 0 (0.0) | 24 (33.8) | - |  |  |
|  | Isolated IGT | 0 (0.0) | 32 (45.1) | - |  |  |
|  | IFG and IGT | 0 (0.0) | 15 (21.1) | - |  |  |
| DM during follow-up (%) | non-DM | 249 (90.2) | 36 (50.7) | - | <0.001 | - |
|  | DM | 27 (9.8) | 35 (49.3) | - |  |  |
| C-peptide (pmol/L) | | 356.79 [275.67, 486.02] | 463.79 [310.18, 617.50] | 620.76 [425.68, 861.67] | 0.002 | <0.001 |
| HOMA2 %B | | 84.65 [73.12, 112.50] | 84.30 [64.20, 99.05] | 52.90 [32.00, 80.20] | 0.037 | <0.001 |
| HOMA2 IR | | 0.78 [0.60, 1.06] | 1.04 [0.70, 1.39] | 1.61 [1.10, 2.30] | <0.001 | <0.001 |
| 1-h plasma glucose (mmol/L) | | 7.77 (2.12) | 10.74 (1.95) | **-** | <0.001 | **-** |
| 2-h plasma glucose (mmol/L) | | 5.54 (1.23) | 8.06 (1.69) | **-** | <0.001 | **-** |

^a^Abbreviations: ACR, albumin creatinine ratio; BHBHK, Better Health for Better Hong Kong; BMI, body mass index; DM, diabetes mellitus; eGFR, estimated glomerular filtration rate; HDLC, High-Density Lipoprotein Cholesterol; HKDR Hong Kong Diabetes Register; HKFDS; Hong Kong Family in Diabetes; IFG, impaired fasting glycaemia; IGT, impaired glucose tolerance; NGT, normal glucose tolerance; TG, triglyceride. ^b^*P** indicates significance for comparison between participants with normal glucose tolerance (NGT) and prediabetes; *P*^†^ indicates significance for comparison across participants people with NGT, prediabetes, or type 2 diabetes.

Table S2. Associations between cardio-metabolic factors and HOMA2 indices in participants with or without diabetes at baseline

|  | **Non-diabetes at baseline** | | | | | | |  | **Diabetes free of insulin treatment at baseline** | | | | | |
| --- | --- | --- | --- | --- | --- | --- | --- | --- | --- | --- | --- | --- | --- | --- |
|  | HOMA2-%B (log2 transformed) | | | HOMA2-IR (log2 transformed) | | | |  | HOMA2-%B (log2 transformed) | | | HOMA2-IR (log2 transformed) | | |
|  | Estimate | SE. | P | | Estimate | SE. | P |  | Estimate | SE. | P | Estimate | SE. | P |
| Age | -0.006 | 0.003 | 0.070 | | 0.001 | 0.004 | 0.797 |  | -0.004 | 0.006 | 0.542 | -0.004 | 0.004 | 0.380 |
| Sex (female) | 0.062 | 0.046 | 0.184 | | -0.079 | 0.061 | 0.199 |  | -0.018 | 0.079 | 0.820 | -0.159 | 0.061 | 0.009 |
| Duration of diabetes (year) | - | - | - | | - | - | - |  | -0.056 | 0.010 | <0.001 | -0.004 | 0.008 | 0.633 |
| Fasting plasma glucose (mmol/L) | -0.439 | 0.045 | <0.001 | | 0.271 | 0.065 | <0.001 |  | -0.268 | 0.008 | <0.001 | 0.065 | 0.010 | <0.001 |
| 1-h plasma glucose (mmol/L) | -0.013 | 0.01 | 0.190 | | 0.053 | 0.013 | <0.001 |  | - | - | - | - | - | - |
| 2-h plasma glucose (mmol/L) | 0.014 | 0.014 | 0.308 | | 0.089 | 0.018 | <0.001 |  | - | - | - | - | - | - |
| HbA1c (%) | - | - | - | | - | - | - |  | -0.319 | 0.018 | <0.001 | 0.097 | 0.017 | <0.001 |
| Body mass index (kg/m^2^) | 0.016 | 0.007 | 0.018 | | 0.055 | 0.008 | <0.001 |  | 0.058 | 0.009 | <0.001 | 0.061 | 0.006 | <0.001 |
| Waist circumference | 0.011 | 0.003 | <0.001 | | 0.028 | 0.003 | <0.001 |  | 0.021 | 0.004 | <0.001 | 0.030 | 0.003 | <0.001 |
| Waist-hip-ratio | 1.204 | 0.417 | 0.004 | | 3.077 | 0.529 | <0.001 |  | 2.128 | 0.700 | 0.002 | 4.586 | 0.508 | <0.001 |
| Systolic blood pressure (mmHg) | 0.001 | 0.002 | 0.422 | | 0.008 | 0.002 | <0.001 |  | -0.002 | 0.002 | 0.389 | 0.007 | 0.002 | <0.001 |
| Diastolic blood pressure (mmHg) | 0.001 | 0.002 | 0.829 | | 0.009 | 0.003 | 0.002 |  | -0.005 | 0.004 | 0.204 | 0.011 | 0.003 | <0.001 |
| Total cholesterol (mmol/L) | 0.007 | 0.027 | 0.800 | | 0.026 | 0.036 | 0.473 |  | -0.215 | 0.039 | <0.001 | 0.068 | 0.030 | 0.026 |
| Triglycerides (mmol/L)* | 0.188 | 0.046 | <0.001 | | 0.356 | 0.059 | <0.001 |  | 0.016 | 0.064 | 0.808 | 0.421 | 0.046 | <0.001 |
| HDL-cholesterol (mmol/L) | -0.153 | 0.057 | 0.008 | | -0.319 | 0.074 | <0.001 |  | -0.279 | 0.123 | 0.024 | -0.438 | 0.093 | <0.001 |
| LDL-cholesterol (mmol/L) | 0.005 | 0.029 | 0.873 | | 0.043 | 0.039 | 0.270 |  | -0.243 | 0.046 | <0.001 | 0.009 | 0.036 | 0.807 |
| Ratio of TG to HDLc* | 0.151 | 0.035 | <0.001 | | 0.298 | 0.045 | <0.001 |  | -0.048 | 0.054 | 0.372 | 0.349 | 0.039 | <0.001 |

^a^Linear regression was used to assess the associations. ^b^Beta coefficient estimates was adjusted for age and sex for participants without diabetes at baseline, and adjusted for age, sex and duration of diabetes for participants with diabetes at baseline. Triglycerides and ratio of TG to HDLc were natural logarithmically transformed due to non-normal distribution.

Table S3. Baseline characteristics of participants stratified by gender in community-based BHBHK-HKFDS and clinic-based HKDR cohorts

|  | | BHBHK-HKFDS | | HKDR | |  |  |
| --- | --- | --- | --- | --- | --- | --- | --- |
|  | | Female | Male | Female | Male | ***P*^*^** | ***P*^#^** |
| Number | | 190 | 157 | 287 | 322 |  |  |
| Age (years) | | 39.67 (7.00) | 40.71 (6.86) | 41.75 (7.19) | 42.38 (6.76) | 0.163 | 0.272 |
| Duration of diabetes (year) | | - | - | 2.00 [1.00, 6.00] | 1.00 [0.00, 3.00] | - | 0.004 |
| Body mass index (kg/m^2^) | | 22.94 [20.78, 26.05] | 23.80 [22.38, 26.47] | 25.68 [22.96, 28.93] | 26.17 [23.85, 29.19] | 0.003 | 0.059 |
| Waist circumference (cm) | | 75.54 (8.52) | 84.01 (7.85) | 83.49 (10.46) | 90.81 (10.07) | <0.001 | <0.001 |
| Waist-hip-ratio | | 0.79 (0.06) | 0.88 (0.06) | 0.85 (0.06) | 0.91 (0.05) | <0.001 | <0.001 |
| Fasting plasma glucose (mmol/l) | | 4.86 (0.50) | 5.04 (0.41) | 8.34 (2.95) | 8.43 (3.03) | <0.001 | 0.690 |
| HbA1c (%) | | - | - | 7.20 (1.67) | 7.43 (1.82) | - | 0.100 |
| HbA1c (mmol/mol) | | - | - | 55 (18) | 58 (20) | - | 0.100 |
| Systolic blood pressure (mmHg) | | 113.40 (16.71) | 120.38 (14.26) | 127.10 (17.16) | 126.93 (14.90) | <0.001 | 0.899 |
| Diastolic blood pressure (mmHg) | | 71.31 (11.06) | 78.31 (8.95) | 75.28 (10.10) | 76.56 (10.14) | <0.001 | 0.122 |
| Total cholesterol (mmol/l) | | 4.84 (0.84) | 5.39 (0.96) | 5.14 (0.96) | 5.00 (1.01) | <0.001 | 0.099 |
| Triglycerides (mmol/l) | | 0.82 [0.62, 1.15] | 1.38 [0.91, 1.85] | 1.44 [0.92, 2.03] | 1.59 [1.10, 2.60] | <0.001 | 0.002 |
| HDL-cholesterol (mmol/l) | | 1.62 (0.47) | 1.37 (0.30) | 1.40 (0.36) | 1.21 (0.28) | <0.001 | <0.001 |
| LDL-cholesterol (mmol/l) | | 2.82 (0.78) | 3.35 (0.87) | 2.98 (0.85) | 2.89 (0.86) | <0.001 | 0.198 |
| Ratio of TG to HDLc | | 0.51 [0.36, 0.83] | 1.03 [0.64, 1.47] | 1.08 [0.62, 1.61] | 1.33 [0.87, 2.19] | <0.001 | <0.001 |
| Urinary ACR (mg/mmol/L) | | 0.90 [0.53, 1.80] | 0.50 [0.34, 0.91] | 1.17 [0.58, 3.61] | 1.19 [0.52, 3.60] | <0.001 | 0.462 |
| Glycaemic status (%) | NGT | 150 (78.9) | 126 (80.3) | - |  | 0.265 | - |
|  | Isolated IFG | 10 (5.3) | 14 (8.9) | - |  |  |  |
|  | Isolated IGT | 19 (10.0) | 13 (8.3) | - |  |  |  |
|  | IFG and IGT | 11 (5.8) | 4 (2.5) | - |  |  |  |
| Use of oral glucose lowering drugs (%) | | - | - | 195 (67.9) | 253 (78.6) | - | 0.004 |
| Use of lipid lowering drugs (%) | | - | - | 32 (11.1) | 60 (18.6) | - | 0.014 |
| Use of BP lowering drugs (%) | | - | - | 97 (33.8) | 101 (31.4) | - | 0.580 |
| Use of RAS inhibitors (%) | | - | - | 45 (15.8) | 70 (22.2) | - | 0.061 |
| C-peptide (pmol/L) | | 363.94 [282.21, 505.68] | 388.39 [287.58, 534.14] | 577.06 [383.77, 799.44] | 643.05 [459.75, 910.69] | 0.310 | 0.006 |
| HOMA2-%B | | 86.75 [74.95, 108.15] | 82.00 [67.90, 108.70] | 50.90 [30.00, 78.35] | 54.45 [34.70, 83.15] | 0.140 | 0.177 |
| HOMA2-IR | | 0.78 [0.61, 1.10] | 0.84 [0.62, 1.15] | 1.50 [0.99, 2.17] | 1.69 [1.19, 2.38] | 0.189 | 0.005 |
| 1-h plasma glucose (mmol/L) | | 7.96 (2.48) | 8.89 (2.21) | **-** | **-** | <0.001 | **-** |
| 2-h plasma glucose (mmol/L) | | 6.21 (1.79) | 5.87 (1.51) | **-** | **-** | 0.064 | **-** |

^a^Data are expressed as mean (SD) or number (%) median [IQR].

^b^Abbreviations: ACR: albumin creatinine ratio, BP, blood pressure, BHBHK, Better Health for Better Hong Kong; eGFR, estimated glomerular filtration rate; IFG, impaired fasting glycaemia; IGT, impaired glucose tolerance; NGT, normal glucose tolerance; HKDR Hong Kong Diabetes Register; HKFDS, Hong Kong Family in Diabetes; RAS, renin angiotensin system, TG, triglyceride.

^c^*P** indicates significance for comparison between men and women in the BHBHK-HKFDS cohort; *P*^#^ indicates significance for comparison between male and female in the HKDR cohort.

Table S4. Logistic regression of HOMA2 %B and HOMA2 IR for progression to type 2 diabetes

|  | Event/Total No. | Unadjusted model | | Model 2 | | Model 3 | | Model 4 | |
| --- | --- | --- | --- | --- | --- | --- | --- | --- | --- |
| Variables |  | OR (95% CI) | P | OR (95% CI) | P | OR (95% CI) | P | OR (95% CI) | P |
| **As per 1 unit of Log2(HOMA2-%B)** |  | 0.77(0.40, 1.45) | 0.417 | 0.02(0.01, 0.08) | <0.001 | 0.03(0.01, 0.10) | <0.001 | 0.09(0.20, 0.39) | 0.002 |
| **HOMA2 %B categorized by median value** |  |  |  |  |  |  |  |  |  |
| Above median value (non-ID) | 29/175 | reference | | | | | | | |
| Below median value (ID) | 33/172 | 0.84(0.48, 1.45) | 0.525 | 3.76(1.68, 8.75) | 0.002 | 3.29(1.43, 7.90) | 0.006 | 1.83(0.72, 4.78) | 0.210 |
| **HOMA2 %B categorized in tertiles** |  |  |  |  |  |  |  |  |  |
| Highest tertile | 16/115 | reference | | | | | | | |
| Middle tertile | 28/116 | 1.97(1.01, 3.95) | 0.050 | 7.21(3.13, 17.67) | <0.001 | 7.35(3.07, 18.88) | <0.001 | 3.95(1.51, 10.93) | 0.006 |
| Lowest tertile | 18/116 | 1.14(0.55, 2.38) | 0.731 | 16.26(5.21, 54.66) | <0.001 | 15.09(4.64, 52.98) | <0.001 | 6.54(1.75, 25.95) | 0.006 |
|  |  |  |  |  |  |  |  |  |  |
| **As per 1 unit of Log2(HOMA2-IR)** |  | 3.26(1.97, 5.54) | <0.001 | 30.61(12.17,85.21) | <0.001 | 19.36(7.29, 56.68) | <0.001 | 8.11(2.59, 27.55) | <0.001 |
| **HOMA2 IR categorized by median value** |  |  |  |  |  |  |  |  |  |
| Below median value (non-IR) | 17/176 | reference | | | | | | | |
| Above median value (IR) | 45/171 | 3.34(1.86, 6.26) | <0.001 | 10.61(4.75, 25.08) | <0.001 | 7.66(3.24, 19.15) | <0.001 | 3.64(1.61, 9.71) | 0.008 |
| **HOMA2 IR categorized in tertiles** |  |  |  |  |  |  |  |  |  |
| Lowest tertile | 9/116 | reference | | | | | | | |
| Middle tertile | 20/116 | 2.48(1.10, 5.96) | 0.033 | 6.78(2.71, 18.40) | <0.001 | 5.04(1.92, 14.29) | 0.001 | 3.72(1.33, 11.16) | 0.015 |
| Highest tertile | 33/115 | 4.78(2.25, 11.14) | <0.001 | 55.22(16.61, 206.23) | <0.001 | 24.62(6.76, 99.33) | <0.001 | 9.09(2.15, 41.49) | 0.003 |

^a^Model 2 was adjusted for age, sex; model 3 was adjusted for variables in model 2 and body mass index, ln(ratio of triglycerides to HDLC), and family history of type 2 diabetes; model 4 was adjusted for variables in model 3 and glycaemic status and plasma glucose at 60 min during 75 gram OGTT. HOMA2-%B and HOMA2-IR were mutually adjusted.

^b^There were increased risks of T2D from the highest tertile to the lowest tertile of HOMA2-%B, and from the lowest to the highest tertile of HOMA2-IR. HOMA2-IR showed greater effect on the risks of progression to T2D independent of other metabolic risk factors, glycaemic status and 1h-PG.

Table S5. Statistics of detection of additive interaction between HOMA2 %B and HOMA2 IR in the association with progression to type 2 diabetes and glycemic deterioration.

|  |  | RERI | AP | S |
| --- | --- | --- | --- | --- |
| Risk of progression to type 2 diabetes | Unadjusted model | 4.80(-4.72, 14.32) | 0.43(-0.02, 0.89) | 1.90(0.74, 4.90) |
|  | Model 2 | 4.83(-4.62, 14.29) | 0.44(-0.005, 0.89) | 1.96(0.74, 5.14) |
|  | Model 3 | 3.01(-3.52, 9.54) | 0.39(-0.15, 0.94) | 1.83(0.58, 5.80) |
|  | Model 4 | 0.36(-2.85, 3.58) | 0.11(-0.86, 1.08) | 1.19(0.22, 6.38) |
| Risk of glycemic deterioration | Unadjusted model | 0.47(-0.84, 1.78) | 0.10(-0.17, 0.37) | 1.14(0.78, 1.68) |
|  | Model 2 | -0.009(-1.36, 1.34) | -0.002(-0.31, 0.30) | 1.00(0.68, 1.47) |
|  | Model 3 | -0.69(-1.69, 0.32) | -0.33(-0.81, 0.15) | 0.61(0.33, 1.13) |
|  | Model 4 | -0.69(-1.72, 0.34) | -0.31(-0.78, 0.16) | 0.64(0.36, 1.15) |

^a^Odds ratios from logistic regression were used for calculating measures of additive interaction for risk of incident type 2 diabetes. Hazard ratios from Cox proportional regression were used for calculating measures for risk of progression to insulin requirement.

^b^Abbreviations: RERI, relative excess risk due to interaction; AP, attributable proportion; S, synergy index. None of these were significant with the 95% confidence interval including the null values which are zero for RERI and AP and one for S.

Table S6. Cox regression of HOMA2-%B and HOMA2-IR for progression to glycemic deterioration

|  | Event/Total No. | Unadjusted model | | Model 2 | | Model 3 | | Model 4 | |
| --- | --- | --- | --- | --- | --- | --- | --- | --- | --- |
| Variables |  | HR (95% CI) | P | HR (95% CI) | P | HR (95% CI) | P | HR (95% CI) | P |
| **As per 1 unit of Log2(HOMA2-%B)** |  | 0.67 (0.59-0.76) | <0.001 | 0.60 (0.53-0.68) | <0.001 | 0.70 (0.58-0.85) | <0.001 | 0.69 (0.57-0.84) | <0.001 |
| **HOMA2-%B categorized by median value** |  |  |  |  |  |  |  |  |  |
| Above median value (non-ID) | 113/304 | reference | | | | | | | |
| Below median value (ID) | 178/305 | 1.83 (1.44-2.32) | <0.001 | 2.21 (1.72-2.85) | <0.001 | 1.57 (1.16-2.13) | 0.004 | 1.64 (1.21-2.22) | 0.002 |
| **HOMA2 %B categorized in tertiles** |  |  |  |  |  |  |  |  |  |
| Highest tertile | 73/203 | reference | | | | | | | |
| Middlle tertile | 95/203 | 1.24 (0.92-1.69) | 0.161 | 1.47 (1.07-2.02) | <0.001 | 1.20 (0.85-1.69) | 0.292 | 1.22 (0.86-1.72) | 0.267 |
| Lowest tertile | 123/203 | 2.03 (1.52-2.72) | <0.001 | 2.78 (2.04-3.80) | <0.001 | 1.79 (1.20-1.68) | 0.004 | 1.83 (1.23-2.74) | 0.003 |
|  |  |  |  |  |  |  |  |  |  |
| **As per 1 unit of Log2(HOMA2-IR)** |  | 1.52 (1.30-1.79) | <0.001 | 1.86 (1.57-2.20) | <0.001 | 1.39 (1.13-1.71) | 0.002 | 1.39 (1.13-1.71) | 0.002 |
| **HOMA2-IR categorized by median value** |  |  |  |  |  |  |  |  |  |
| Below median value (non-IR) | 127/309 | reference | | | | | | | |
| Above median value (IR) | 164/300 | 1.71 (1.35-2.16) | <0.001 | 2.07 (1.62-2.65) | <0.001 | 1.39 (1.03-1.86) | 0.029 | 1.40 (1.05-1.88) | 0.023 |
| **HOMA2-IR categorized in tertiles** |  |  |  |  |  |  |  |  |  |
| Lowest tertile | 82/203 | reference | | | | | | | |
| Middle tertile | 94/203 | 1.25 (0.93-1.68) | 0.145 | 1.63 (1.20-2.21) | 0.002 | 1.33 (0.96-1.84) | 0.084 | 1.37 (0.99-1.91) | 0.058 |
| Highest tertile | 115/203 | 1.84 (1.38-2.45) | <0.001 | 2.58 (1.91-3.50) | <0.001 | 1.53 (1.07-2.20) | 0.021 | 1.57 (1.10-2.26) | 0.014 |

^a^Model 2 was adjusted for age, sex, and strata by disease duration; model 3 was adjusted for variables in model 2 plus ln(TG/HDLc ratio), body mass index and systolic blood pressure, and strata by HbA1c; model 4 was adjusted for variables in model 3 plus baseline treatment with oral glucose lowering drugs. HOMA2-%B and HOMA2-IR were mutually adjusted.

^b^When stratified participants into tertiles, the lowest tertile of HOMA2-%B and the highest tertile of HOMA2-IR were independently associated with increased hazards of glycemic deterioration controlling for metabolic factors and baseline treatment of OGLDs. HOMA2-%B showed greater effect on the risks of glycemic deterioration.

**Supplement Figures**

Figure S1. Flowchart of cohorts selection


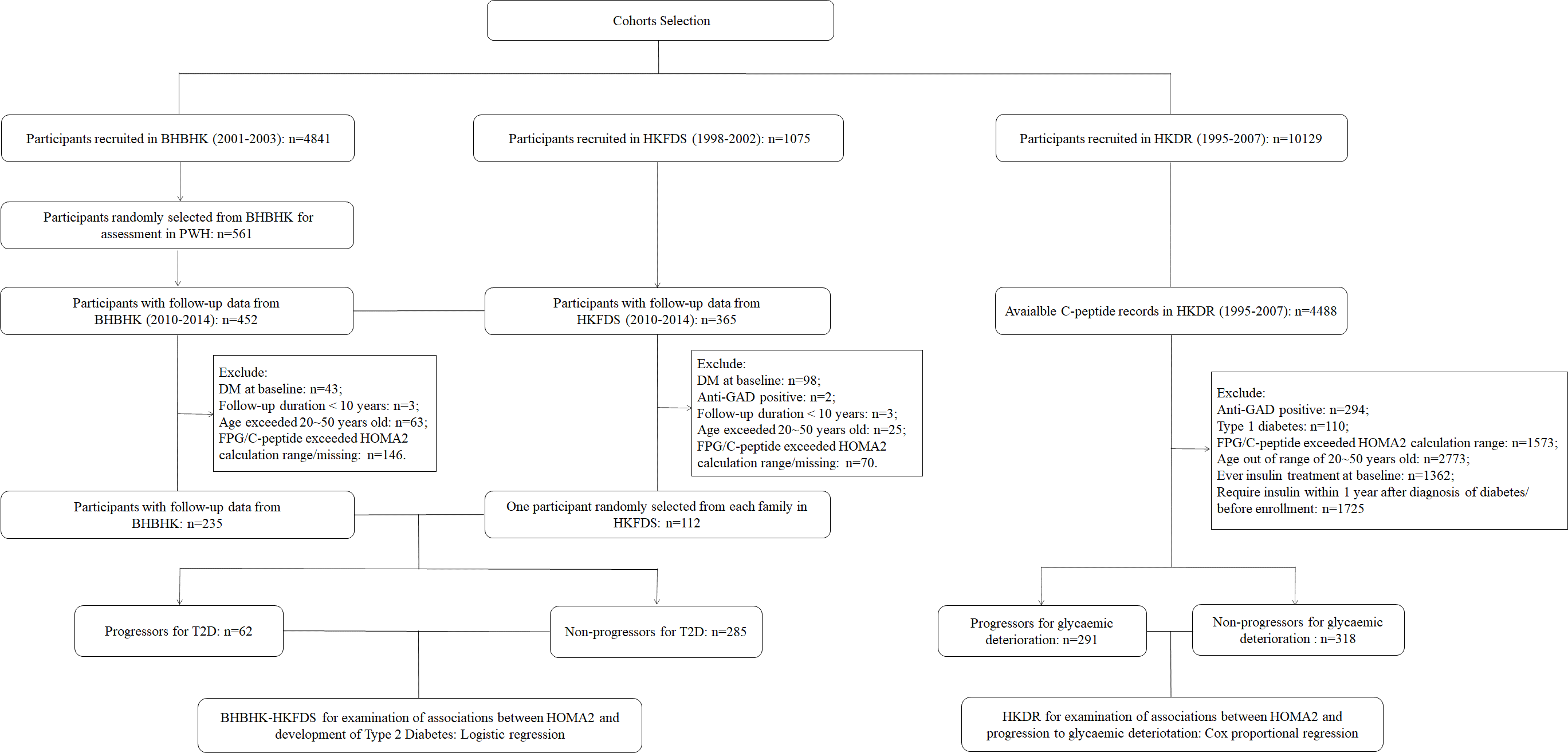


Figure S2. Relationship between baseline HOMA2-%B and HOMA2-%S in progressors versus non-progressors for type 2 diabetes (T2D) or glycaemic deterioration.


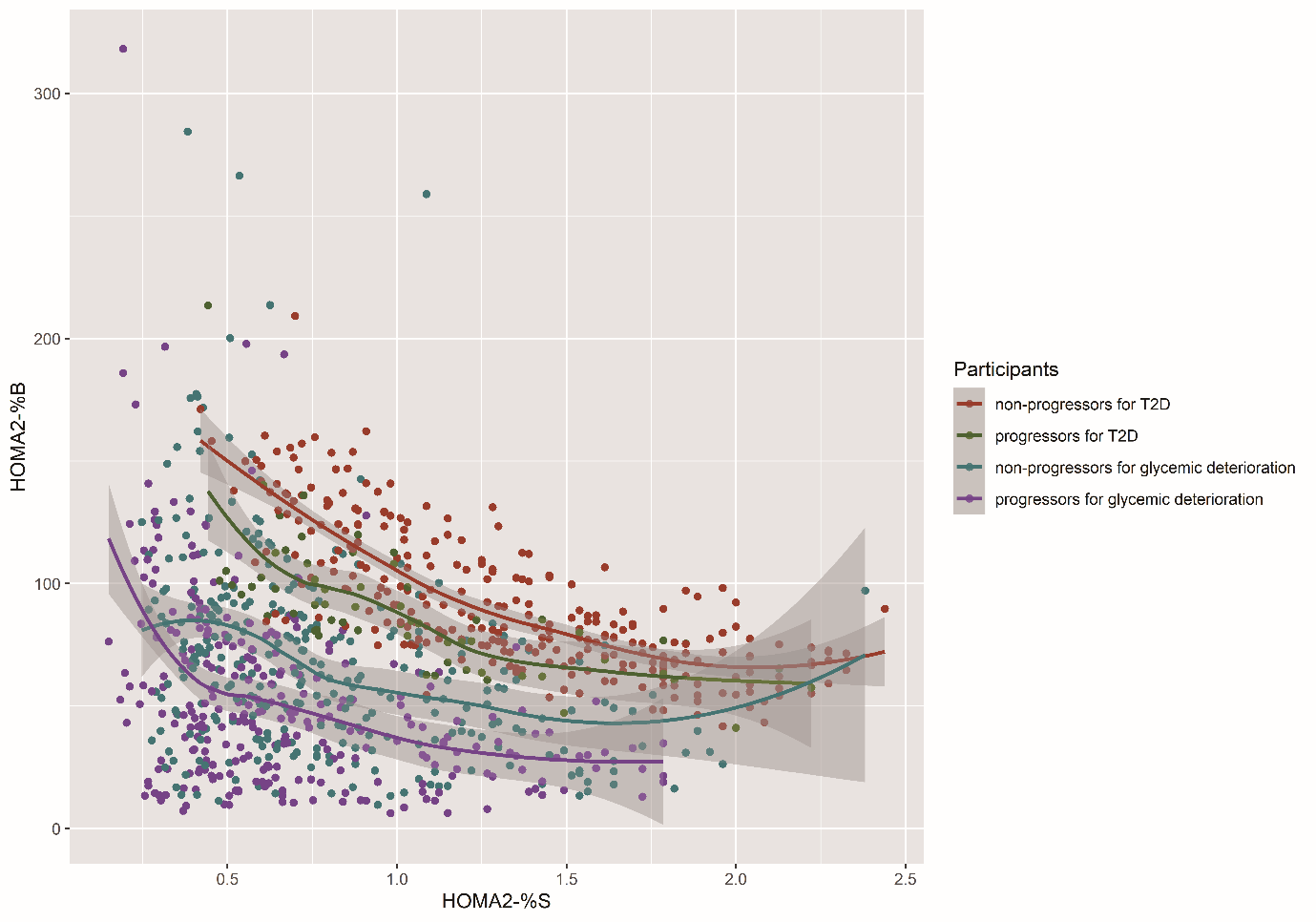


Footnote:

^a^HOMA2-%S was calculated by the reciprocal of HOMA2-IR, as an index of insulin sensitivity.

^b^Shaded area indicates the 95% confidence interval of the smooth lines.

Figure S3. Logistic regression for progression to type 2 diabetes stratified by the median values of HOMA2 %B and HOMA2 IR respectively


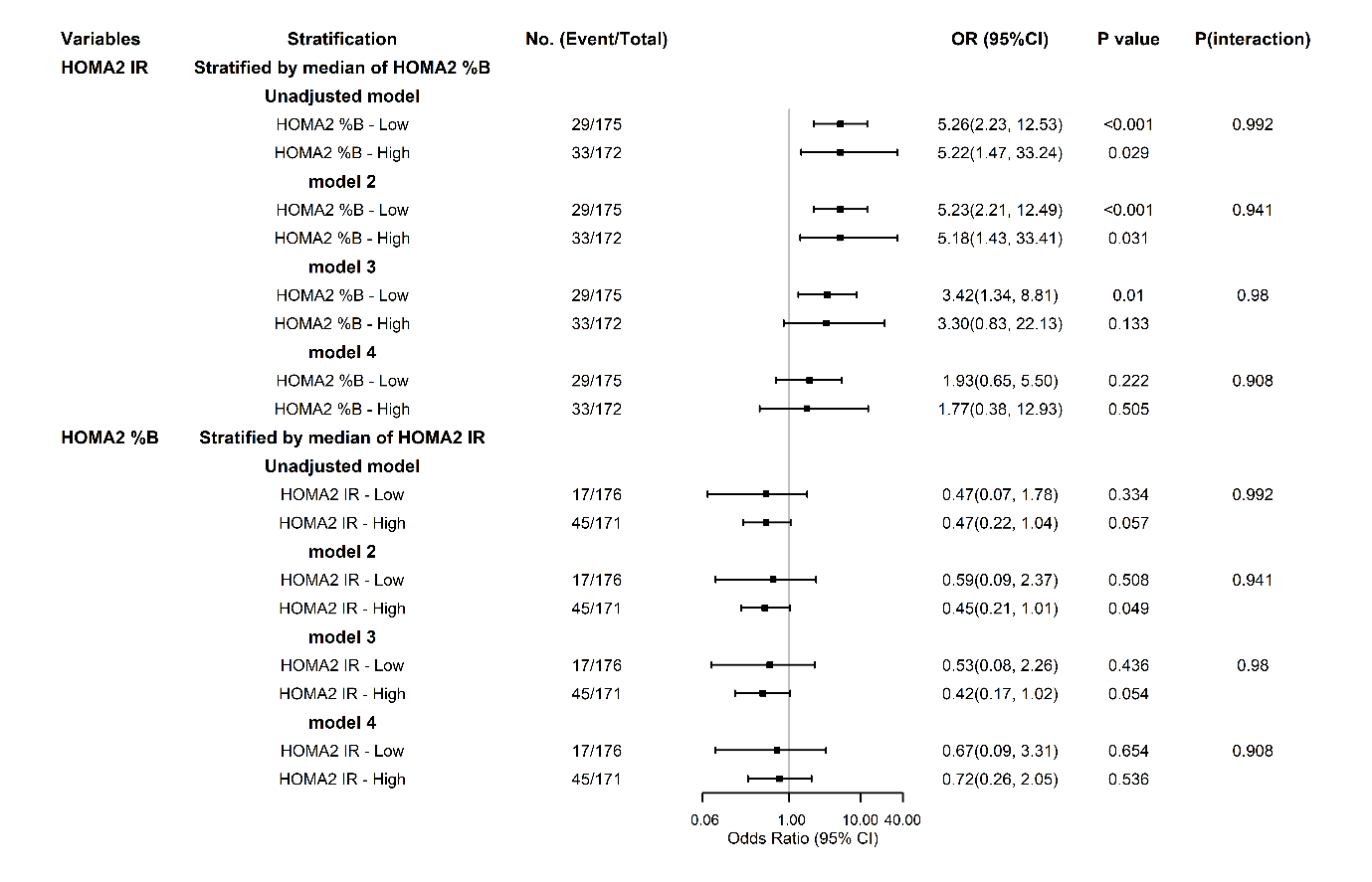


Footnote: ^a^Model 2 was adjusted for age, sex; model 3 was adjusted for variables in model 2 plus body mass index, ln(ratio of triglycerides to HDL-C), and family history of type 2 diabetes; model 4 was adjusted for variables in model 3 plus glycaemic status and plasma glucose at 60 min during 75 gram oral glucose tolerance test.

Figure S4. Survival curve of glycemic deterioration stratified by median or tertile values of HOMA2 %B and HOMA2-IR


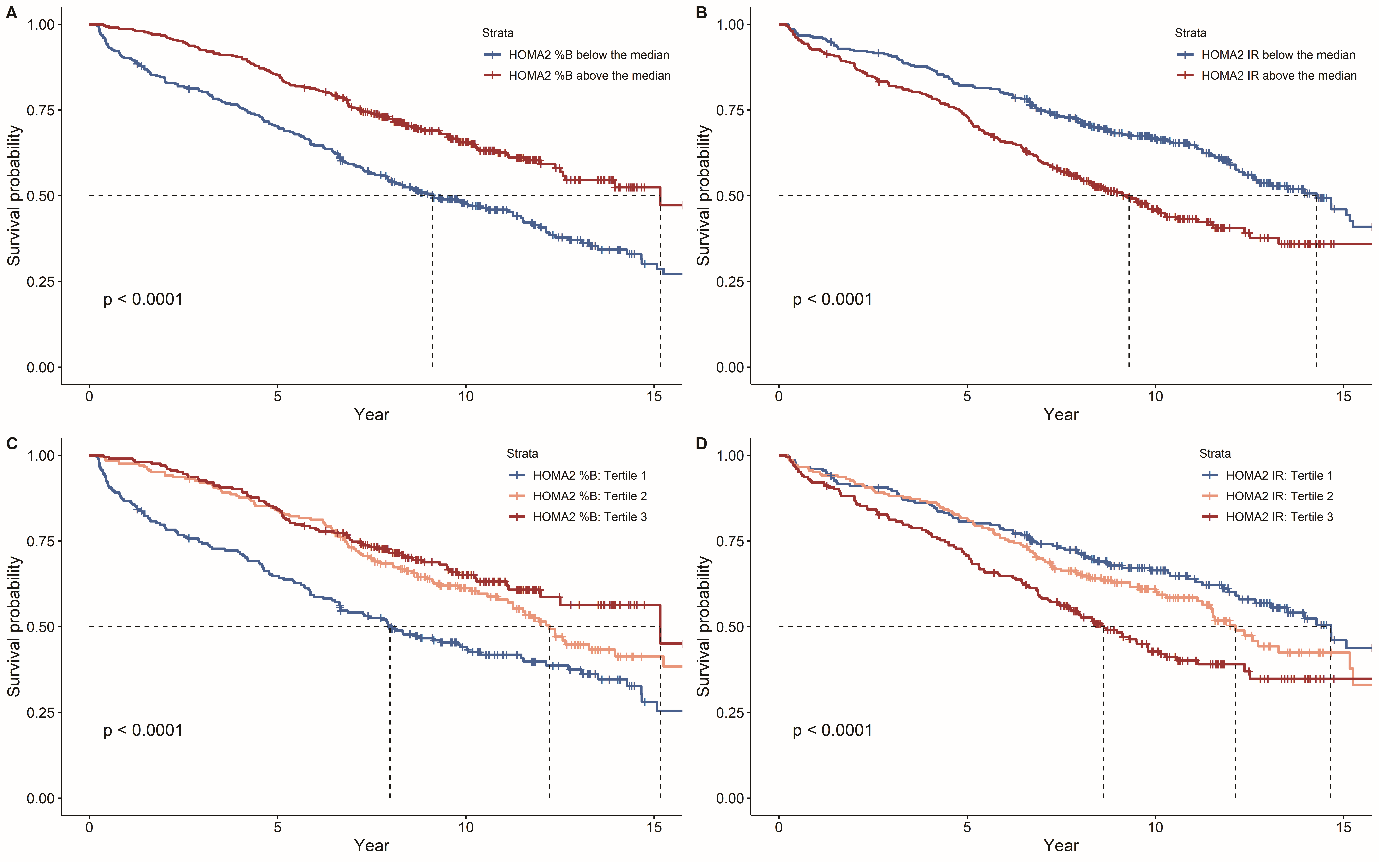


Footnote: ^a^Survival curves of progression to glycemic deterioration in patients with type 2 diabetes, stratified by the median value of HOMA2-%B (A) or HOMA2-IR (B) and tertile value of HOMA2-%B (C) or HOMA2-IR (D). ^*^P-value was calculated by log-rank test.

Figure S5. Cox regression for glycemic deterioration stratified by the median value of HOMA2 %B and HOMA2 IR respectively


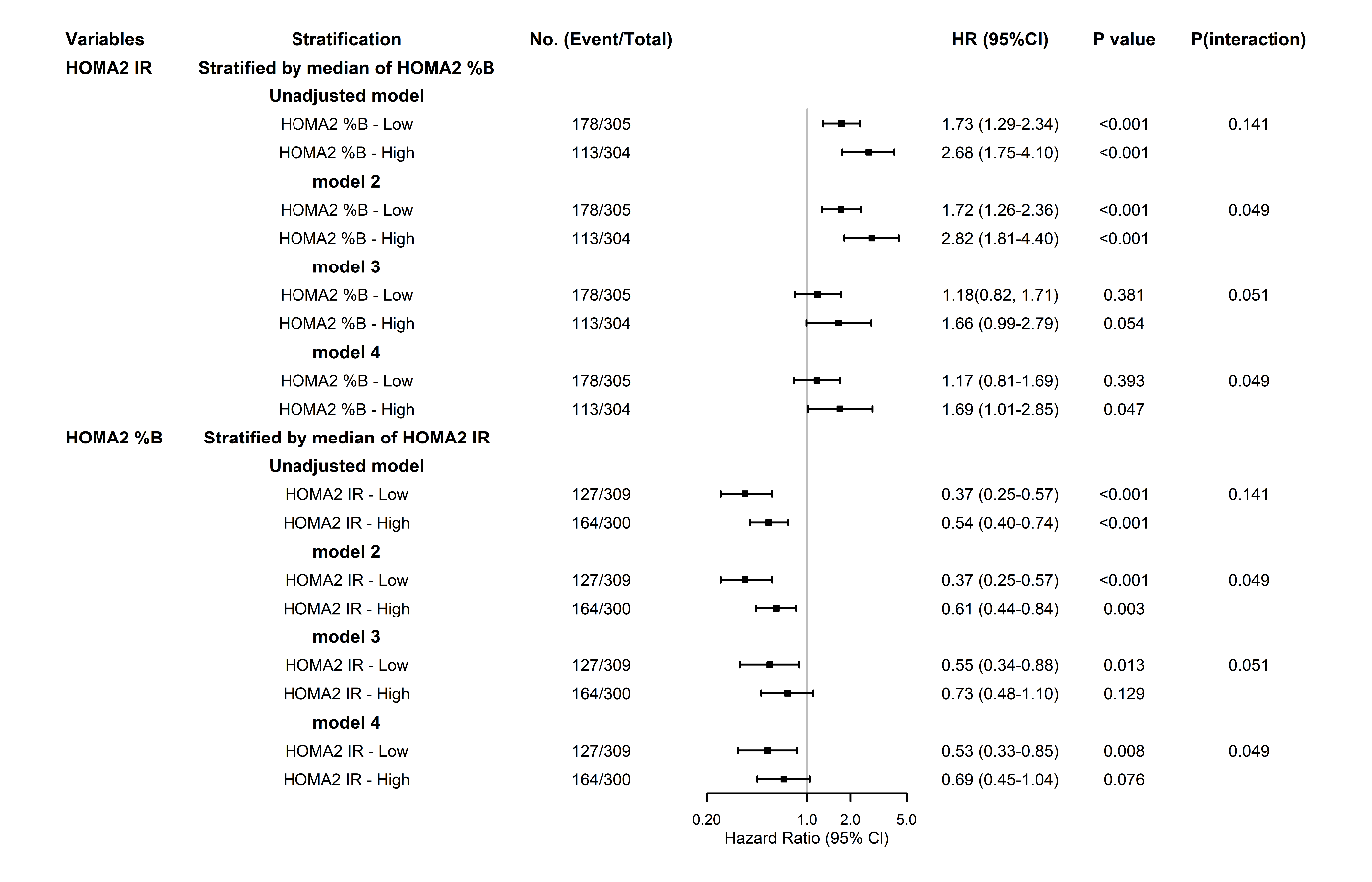


Footnote: ^a^Model 2 was adjusted for age, sex, and strata by disease duration; model 3 was adjusted for variables in model 2 plus ln(TG/HDLc ratio), body mass index and systolic blood pressure, and strata by HbA1c; model 4 was adjusted for variables in model 3 plus baseline treatment of oral glucose lowering drugs.

Figure S6. Receiver Operating Characteristic Curve of HOMA2 indexes on onset of type 2 diabetes.

A. B.


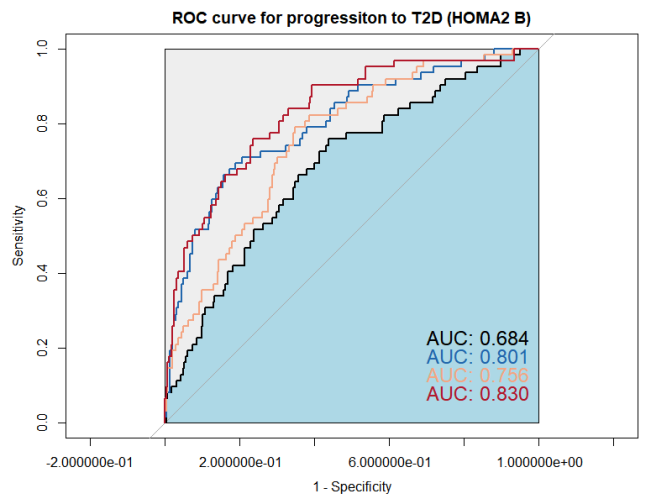

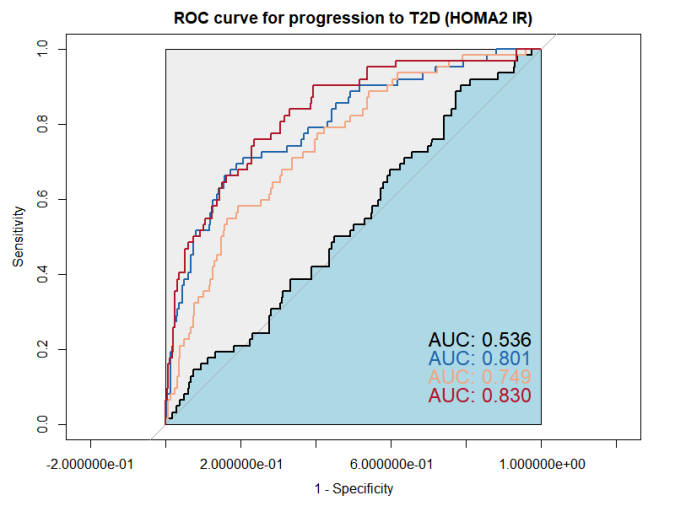


Footnote: ^a^In figure S6, black line refers to basic model (model 1) including age and sex for incident T2D, blue line refers to model 2 adding logarithmically transformed HOMA2 indexes on model 1, yellow line refers to model3 adjusted for cardio-metabolic risk factors and family history, red line refers to model 4 adding logarithmically transformed HOMA2 indexes on model 4. In these models, HOMA2-%B and HOMA2-IR were mutually adjusted.

^c^In Figure S6A, *p*(ΔAUC) for model 1 < 0.001, *p*(ΔAUC) for model 2 < 0.001; In Figure S6B, *p*(ΔAUC) for model 1 < 0.001, *p*(ΔAUC) for model 2 = 0.0.002.
